# In situ multimodal imaging reveals compartmentalization of reovirus replication factories

**DOI:** 10.1099/jgv.0.002350

**Published:** 2026-10-27

**Authors:** Eva Ďurinová, Tomas Bily, František Kitzberger, Zdeněk Franta, Peter Mojzeš, Petr Chlanda, Roman Tuma

**Affiliations:** 1 Faculty of Science, University of South Bohemia in České Budějovice, České Budějovice, Czechia; 2 Biology Centre, Czech Academy of Sciences, České Budějovice, Czech Republic; 3 Faculty of Mathematics and Physics, Charles University, Ke Karlovu 5, Prague 2, Czech Republic; 4 Department of Infectious Diseases, Virology, Heidelberg University, Heidelberg, Germany; 5 BioQuant Center for Quantitative Biology, Heidelberg University, Heidelberg, Germany

**Keywords:** avian reovirus, inclusion bodies, phase separation, viroplasms

## Abstract

Avian reovirus (ARV) belongs to *Reoviridae* family of viruses with segmented dsRNA genomes. ARV is responsible for significant economic losses to the poultry industry by causing tenosynovitis and chronic respiratory disease in chickens. ARV virion exhibits an outer protein shell enclosing an inner core, which contains 10 unique dsRNA segments and multiple copies of viral polymerase. ARV assembly takes place in so-called viral factories (VFs). They are cytoplasmic membrane-less inclusions, which accumulate viral proteins and RNA and assist capsid assembly, genome packaging and replication. VFs also provide protection from the host cell defence mechanisms by sequestering viral RNA. Given the dense nature of VFs, little is known about ARV assembly and packaging intermediates. To determine the architecture of VFs across the different length scales and infection course, we employed a wide range of imaging methods. Our data reveal that spherical and fluid VF enriched in viral protein σNS become surrounded by σNS-depleted solid-like compartments at late stages of infection. We demonstrate that confocal Raman microscopy allows label-free imaging of VF composition. Volume electron microscopy, along with electron tomography (ET) and *in situ* cryo-ET, revealed that the inner phase of VF concentrates viral non-structural proteins and assists assembly of viral cores. The larger, RNA-filled and double-layered particles are assembled at the periphery of the inner fluid phase of VF, forming ordered virion arrays. This suggests that ARV VFs are functionally compartmentalized: the inner fluid phase assists RNA assortment and core particle assembly, whereas the peripheral phase is the site of RNA replication inside viral cores and virion completion.

## Data Summary

All supporting data, code and protocols have been provided in the article or through supplementary data files. Dataset with supplementary videos isare available through the Microbiology Society’s data repository Figshare account: https://doi.org/10.6084/m9.figshare.31959531 [1]. Imaging files were deposited in BioImage Archive (accession number S-BIAD3794) and MicrobioRaman database (accession number S-MBRS39).

## Introduction

Avian (ortho)reovirus (ARV) belongs to the large family of *Reoviridae*, which has a wide host range from fungi to vertebrates [2]. *Reoviridae* include serious human pathogens such as rotaviruses (RVs) causing life-threatening diarrhoeal diseases in infants and young children [3], significant plant pathogens such as the rice dwarf virus [4] and viruses causing important livestock and poultry diseases (e.g. bluetongue virus (BTV) [5] and ARV [6, 7]). ARV inflicts significant economic losses on the worldwide poultry industry, mainly by causing viral arthritis (tenosynovitis), and the available vaccines fail to protect against newly emerging strains [8]. An alternative treatment based on interference with the viral replication cycle would offer a new way to tackle this problem.

ARV has icosahedral virion composed of the inner core (T=1, proteins λA, λB, λC, μA, σA) containing 10 dsRNA segments and enclosed by an outer capsid layer (T=13, proteins μB and its proteolytic fragments µBC and µBN, and σB, σC) of diameter 85 nm [9] (Fig. S1, available in the online Supplementary Material). The virion has 12 pentameric turrets (protein λC) protruding at the icosahedral fivefold vertices from the inner core through the outer layer [9] which constitute the capping enzyme [10]. Like other reoviruses, the inner core contains the RNA-dependent RNA polymerase (λB [11]). Protein σC, which emanates from the fivefold vertices, is responsible for cell attachment and its fold is related to that of adenovirus fibre [12]. ARV structure is closely related to that of mammalian reovirus (MRV) for which a recent asymmetric reconstruction was obtained [13]. All of the 10 RNA segments are essential for the virus replication and are translated into 12 primary protein products, 8 structural proteins of the virion and 4 non-structural (NS) proteins that are involved in virus replication, morphogenesis and cell fusion [14–16].

The gene expression and replication of ARV have not been described in detail, but a framework can be inferred from analogy with other dsRNA viruses [17]. The negative strands of the 10 dsRNA genome segments are used as templates for mRNA synthesis by the dsRNA-dependent-RNA-polymerase within the core, including addition of a cap at the 5′ end when leaving the particle through the turret. The mRNA molecules are used to synthesize viral proteins at the ribosomes in the cytoplasm but also serve as precursors for genomic RNA. ARV replication and assembly take place in so-called viroplasm or viral factories (VF) [18] (Fig. S1). VFs are a common feature of *Reoviridae*, representing dense cytoplasmic inclusions where viral particles are assembled and the viral genome is packaged and replicated [19] and are often surrounded by arrays of mature virions [20, 21] and/or interact or nucleate from lipid droplets, as shown for RV [22, 23]. ARV VFs are globular, membrane-less neo-organelles and, unlike its MRV counterpart, are not associated with the microtubule network [24].

The main component of VFs, μNS protein, is sufficient for the formation of similar inclusions in heterologous (non-infected) cells and recruits other viral proteins [18, 25]. Another major component is the RNA chaperone σNS, which is thought to protect ssRNA and assist the formation of specific RNA–RNA interactions during genomic segment selection [26, 27]. This latter process is poorly understood for ARV but evidence obtained for RV and BTV suggests that specific base-pairing between single-stranded genomic RNA precursors mediates RNA segment selection across all members of the *Reoviridae* [28–31] and is mediated by the NS RNA binding chaperones (RV NSP2 [32] NS2 in BTV [33]). Recently, VFs of several *Reoviridae* members, including ARV, were shown to be biological condensates formed by liquid–liquid phase separation (LLPS) [34, 35]. Super-resolution imaging suggested that RV VFs, despite being fluid, may not be homogeneous but exhibit gradients of viral proteins [36] and may undergo further phase transitions to a gel-like (solid) phase [19]. MRV was shown to assemble in two stages, during which the core particles are assembled first in early VFs, while the outer layer and maturation happen in late VFs that are located within the peri-nuclear space and in the vicinity of lipid droplets and endoplasmic reticulum (ER) membrane [37].

Recent electron microscopy provided snapshots of cells or cell lysates infected by MRV [21], RV [38] and BTV [39], which revealed structures of possible assembly intermediates. Indented single-layered particles (SLPs) were found prominent among the intermediates, suggesting that RNA is being packaged into these empty particles in a fashion similar to the dsRNA bacteriophages of the *Cystoviridae* family [40, 41], possibly with assistance of viral capsid proteins (VP6 in BTV [39]). These studies focused on obtaining medium- to high-resolution reconstructions from frozen cell snapshots and thus on the cellular context (e.g. association with lipid droplets and other structures). However, phase separation and compartmentalization changes in the course of infection were not considered.

Here, we follow ARV VF morphogenesis and virus production within the cellular context using multiscale and multimodal imaging of infected cells. VFs form independently from lipid droplets and remain fluid in later stages of infection, suggesting VFs are stable systems. However, VF mobility becomes restricted in later stages of infection by being embedded in viral arrays (VAs) of newly assembled virions. Confocal Raman microscopy (CRM) imaging revealed that VFs are protein-rich condensates with a density resembling that of the nucleolus and are void of lipids. VAs have a biomolecular composition and density similar to that of nucleoplasm and are interspersed with lipid membranes belonging to the ER network. Electron microscopy revealed that SLP assembly takes place within the VF interior from which virions seem to be excluded, and genome packaging and replication may take place at the VF–VA boundary. Thus, ARV assembly seems compartmentalized by principles of LLPS without the need for lipid droplets or other membrane structures. However, during later infection stages, fusion of infected cells and accumulation of viruses cause VA to invade spaces within ER networks. In summary, while ARV, a representative of the fusogenic orthoreovirus group [2], shares basic features of LLPS-based VFs, it does not rely on lipid and cytoskeletal structures for assembly, a feature typical of orthoreoviruses belonging to the non-fusogenic group, such as MRV.

## Methods

### Cell culture

ARV infection was studied in the mammalian cell line BHK-21 (baby hamster kidney) that is susceptible to ARV strain S1133 infection [42] and simultaneously suitable for microscopy imaging (well-adherent, fast-growing). We developed a stable cell line of BHK-21 expressing C-terminally fluorescently (mCherry)-tagged viral non-structural protein σNS [35]. We followed the protocol for cell growth and transfection as described in [35]. The σNS construct was chosen for transfection instead of μNS due to the ability of μNS to form VF-like inclusions by itself, which would interfere with observation of true VFs during infection. On the other hand, uninfected σNS–mCherry-expressing cells show only a diffuse fluorescent signal in the cytoplasm, while upon infection, the cytoplasmic pool of σNS–mCherry is recruited into VFs by virus-encoded, unlabelled μNS.

### Live-cell imaging

Live-cell imaging was performed using 3D Cell Explorer (Nanolive), combining epifluorescence and refractive-index-contrast holotomography. Holotomographic microscopy reconstructs three-dimensional (3D) images by measuring optical phase shifts caused by variations in cellular refractive index (RI), using holographic illumination from multiple angles. The resulting 3D RI maps are roughly proportional to the local mass density and provide label-free information on the spatial distribution and morphology of intracellular structures. The RI maps can be subjected to 3D volume segmentation in a similar fashion as any other density maps and correlated with the epifluorescence signals for biological interpretation (see [35] for methodological details).

The cells were seeded into 35 mm dishes with a glass bottom (ibidi, μ-Dish), 2–3×10^5^ cells per dish, in 2 ml of FluoroBrite DMEM (Gibco). After 24 h, the cells were infected by S1133 vaccine strain. One vial of the lyophilized vaccine was diluted in 1 ml of PBS. After removal of the growth medium, 0.2 ml of the vaccine/PBS solution (multiplicity of infection ~10) was incubated in the dish for 1 h [room temperature (RT), gentle shaking, PBS was added to the total volume of 0.5 ml to avoid drying when constant shaking was not available]. Then the inoculum was removed and 2 ml of fresh FluoroBrite DMEM was added. The cells were further incubated at 37 °C in 5% CO_2_ or directly put in 3D Cell Explorer heated to 37 °C with 5% CO_2_ for live imaging. Series of images were taken at 5 min (or shorter) intervals with the acquisition of both the epifluorescent image (TRITC channel) at the focal plane and a stack of 90 z-slices of RI contrast at each interval. Volumetric and fluorescent datasets were deposited into BioImage archive https://www.ebi.ac.uk/bioimage-archive/ [43].

### Fluorescence recovery after photobleaching

Fluorescence recovery after photobleaching (FRAP) is a method to study the fluidity (diffusion of biomolecules) of cellular compartments, and its application to reovirus infection has been described [35]. Briefly, it uses a high-intensity confocal beam to bleach a spot in the sample and then measures, in a wide-field mode, epifluorescence recovery due to the diffusion of fluorescently labelled molecules into the bleached volume. Our setup consisted of an Olympus IX70 inverted microscope with a 60× Olympus objective (NA 1.49, oil, APO N) and a stage with a chamber supplying CO_2_ and controlling temperature set to 37 °C. The source of both excitation and bleaching light was Toptica Chrome iCLE laser CW engine line 561 nm, with the modification of splitting the beam into two channels – a confocal bleaching and widefield excitation beam – through a polarizing beam splitter (Thorlabs CCM1-PBS251/M). The signal was detected on an EM-CCD camera (Ixon Ultra, Andor). This setup can follow the recovery of multiple moving objects from milliseconds to minutes. The samples for FRAP were prepared in the same way as for the live-cell imaging. The bleached spot was tracked within frames and integrated intensity extracted as a function of time. The time-dependent intensity was then approximated with one (slow, recovery in minutes) or two exponential (fast and slow) terms depending on the goodness of fit, which was judged by the randomness of the residuals. The presence of a fast-recovering component reflects the fact that some fluorescent VFs were smaller than the bleached spot; hence, the recovery signal contained contributions from the surrounding cytoplasm, which recovers considerably faster.

### Sample preparation for transmission electron microscopy by chemical fixation

Samples for transmission electron microscopy [TEM and electron tomography (ET)] were prepared by two protocols. The first used aldehyde for chemical fixation of the sample. The BHK-21 cell culture was grown in a glass-bottom 35 mm dish (ibidi, μ-Dish) and infected by ARV (same protocol as for live cell imaging). After the desired time post-infection, the growth medium was discarded and replaced by a freshly prepared fixing solution (2% formaldehyde, 2.5% glutaraldehyde in 0.15M cacodylate buffer with 2 mM CaCl_2_). The dish with the fixing solution was incubated at 37 °C for 15 min and then stored in a fridge before further steps (no longer than 3 days). The next steps included contrasting by osmium tetroxide (2% solution in buffer, RT, 1 h) and potassium ferrocyanide (2.5% in water, RT, 1 h, no washing steps after osmium tetroxide), incubation in thiocarbohydrazide (1% in water, RT, 1 h) and *en bloc* staining by uranyl acetate (1% in water, 4 °C, overnight). The glass bottom of the dish with the cell monolayer was then removed and transferred into a whole-glass Petri dish. The samples were then dehydrated with increasing concentrations of ethanol up to 100%. The ethanol was then substituted with 100% acetone. The treated samples were infiltrated with resin/acetone solutions until reaching 100% resin (Epon SPI-pon 812, SPI) and stored in a desiccator overnight. Then the glass-bottom dish with the cell monolayer was placed on a rubber stand, covered with a fresh 100% resin and then empty resin blocks were put on the glass, with the cell monolayer between the glass and the empty resin block face. The samples were left to polymerize for 48 h at 60 °C. After polymerization was complete, the glass was removed by several freeze–thaw cycles (boiling water–liquid nitrogen) and the uncovered block facets were trimmed and sectioned to a thickness of 70 nm using an ultramicrotome Leica EM UC 7 (Leica Microsystems) with a diamond knife (Diatome). The sections were put on copper grids mesh 400, stained by uranyl acetate and lead citrate and carbon-coated before observation.

### Sample preparation for TEM by cryo-fixation

The second protocol for sample preparation for TEM was similar to that described above except that the first steps of fixation and *en bloc* contrast which were replaced by high-pressure freezing of the sample on a sapphire disc (the 3 mm discs were put on the bottom of a Petri dish before cell culture was seeded) followed by freeze substitution (the sample was transferred into 2% osmium tetroxide in acetone and slowly heated from −90 to 4 °C, washed in acetone, infiltrated by Epon resin and polymerized).

### TEM and ET imaging at room temperature

We used a Jeol JEM 1400Flash 120kV microscope (JEOL) equipped with CMOS camera Xarosa (Emsis) for TEM data acquisition and ET data acquisition. The data for the serial ET were collected automatically using SerialEM [44] as single-axis tilt series in the range ±60° with increment of 1° (121 images in total) either at 12,000× or 15,000× magnification with a pixel size either of 8.2 or 6.6 Å. The IMOD software package was used for the tomogram reconstructions, stitching and anisotropic diffusion filtering [45].

### Focused ion-beam scanning electron microscopy volume imaging at room temperature

Focused ion-beam scanning electron microscopy (FIB-SEM) samples were prepared similarly to the above-described protocol (TEM chemical fixation), except that they were not cut into ultrathin sections, but the trimmed resin pyramid with the sample on the top was mounted onto a sample holder. Prior to loading into the FIB-SEM microscope, sample grids were coated with platinum sputtering (20 nm) using a Leica ACE600 cryo-sputter coater (Leica Microsystems). Samples were then transferred to a Crossbeam 550 FIB-SEM microscope (Carl Zeiss). The stage was positioned at a working distance of 5.1 mm and tilted to 54° and further protected by a layer of deposited organic platinum assisted with FIB. Serial sectioning of the sample was followed to generate the FIB-SEM volumes using SmartFIB software (Carl Zeiss). Imaging conditions were 1.5 kV acceleration voltage and a probe current of 2 nA using the backscattered electron detector and at a final pixel size of 5 nm. FIB milling was done at 30 kV and 1.5 nA with a sectioning step of 50 nm.

Raw FIB-SEM image stack pre-processing was done using ImageJ. To remove the curtain artefact in the raw images, a vertical band-pass filter between 1 and 300 pixels was applied. Image stack alignment was done using Linear Stack Alignment with SIFT [46]. Denoising of stack images was performed with the Noise2Void plugin on Fiji [47]. Segmentation was done in MIB [48] and the model was visualized in Amira (Thermo Fisher Scientific). An early version of Segment Anything Model 1 with a 2.5 GB backbone was employed as a segmentation accelerator to assist in generating ground-truth annotations for deep learning [49]. These annotations were subsequently used to train a 2D semantic segmentation model in DeepMIB [50] based on a ResNet50 architecture, which was then used to generate an initial prediction for the remainder of the dataset. The predicted segmentation was manually corrected and refined. As this workflow was intended primarily to accelerate annotation and support semi-automatic segmentation, rather than to benchmark a fully automated segmentation model, no quantitative evaluation metrics are provided.

### Cryo-focused ion beam milling

The BHK-21 cells were seeded on Au holey carbon grids in the freshly glow-discharged 35 mm dish with the bottom covered by a silicone elastomer layer (Sylgard 184, DOW Europe). The culture was infected following the same protocol as described in a previous section (Live-cell imaging). The grids were cryo-fixed at 23 hour post-infection (hpi) using EM GP2 automatic plunge freezer (Leica Microsystems).

Cryo-FIB milling was done with Aquilos 2 dual-beam cryo-FIB-SEM (Thermo Fisher Scientific). Grids were mapped by cryo-SEM. The cells were covered with an organo-metallic platinum layer and milled gradually in five steps with a stage angle between 15° and 18° using a gallium ion beam using AutoTEM (Thermo Fisher Scientific). The last two milling steps were performed manually with a nominal thickness of 150 nm. Micro-expansion joints were used to minimize lamella bending [51].

### Cryo-electron tomography

Montages and tilt series were collected on a Titan Krios TEM (Thermo Fisher Scientific) operated at 300 keV and equipped with a BioQuantum^®^ LS energy filter with a slit width of 20 eV and K3 direct electron detector (Gatan Ametek, Inc.) using SerialEM [44]. The tilt series were acquired at 33,000× magnification with a pixel size of 2.671 Å/px at –4 µm defocus, with an electron dose of ~3 e^-^ Å^−2^ per projection. A dose-symmetric acquisition scheme was used with an effective tilt range of +68° to −52° in 3° increments. Tilt series was aligned using patch tracking or fiducials (2–5 nm large metal clusters of Pt and Au that are deposited on lamella during milling) in Etomo [45]. Tomograms were reconstructed using an R-weighted back projection algorithm after 2D CTF correction, dose-weighting filter and application of SIRT-like filter 5 in the IMOD software package [45]. Electron microscopy data sets were deposited into the BioImage archive https://www.ebi.ac.uk/bioimage-archive/ [43].

### Raman microscopy

Raman mapping of cells was done using an upright CRM WITec alpha300 RSA (Oxford Instruments-WITec, Germany) equipped with the 60× water-immersion objective UPlanSApo, NA 1.2 (Olympus, Japan) and optical-fibre-connected spectrographs UHTS300 and UHTS400 (WITec, Germany) with CCD cameras (Oxford Instruments-Andor, Ireland) optimized for detection in the visible and near-infrared regions, respectively. The 532 or 647 nm lasers, with ~20 mW of excitation power at the focal plane, were used. Raman measurement and the workflow for processing the results were previously described in detail elsewhere [35]. The cells were fixed with 3% glutaraldehyde, twice washed with PBS buffer and overlaid with a 1- to 2-mm-thick layer of the 1% low-temperature-melting agarose in PBS and sealed with a 20 mm glass coverslip and CoverGrip sealant (Biotium, USA). This ensured mechanical stability and prevented dehydration during imaging in the upright microscope.

Individual VFs in cells were localized using wide-field σNS–mCherry fluorescence. Since setting the correct position of the VF in the axial direction is subject to a significantly greater error than the estimation of its position in the x-y plane, it was more appropriate to map this object in the axial plane. Raman mapping was therefore preferably performed as an axial z-scan with a scan step of 200 nm in both directions, a voxel size of ~1 µm^3^ and an integration time of 0.07 or 0.10 s per voxel. Typically, an area of 8 µm (in the x-y plane) × 8 µm (in z-direction) was measured. Alternatively, lateral scans (in x-y plane) or z-stacks of several (typically 16) consecutive lateral layers were obtained for the construction of 2D or 3D images of VFs.

In addition to z-scans of individual VFs selected based on their fluorescence, large areas (up to 40 µm × 40 µm) covering the nuclei and their surroundings were also measured, where the presence of VFs and extensive VAs could be assumed based on the electron microscopy imaging of samples cultured in the same way. In this case, z-stacks (usually 16 layers in a stack, with an axial step of 500 nm) were measured and processed together. By processing such z-stacks together, it was possible to construct 3D maps based on Raman chemical contrast. The main disadvantage of this approach is the length of data acquisition (up to 12 h). The advantage is the independence from fluorescent labelling required for targeted selection of the measured structures. All CRM data were deposited into MicrobioRaman open access database https://www.ebi.ac.uk/biostudies/MicrobioRaman/studies [52].

Data were analysed using WITec Project Six Plus ver.6.2 software (Oxford Instruments-WITec, Germany) by implementing the following steps: cosmic ray removal, cropping of the spectral edges affected by detector margins, background subtraction (approximated by a third-order polynomial) and spectral demixing with the True Component Analysis tool (Oxford Instruments-WITec, Germany). In this way, it was possible to generate contrast allowing the identification of cell nuclei, nucleoli, lipid droplets, VF, VA and other intracellular structures. Although some structures could be clearly identified by chemical composition based on spectral decomposition using the True Component Analysis tool, others exhibited very similar chemical compositions and differed only in overall density (e.g. nuclei and nucleoli). For spatial differentiation and identification of some structures, it was therefore necessary to use a combination of intensity and Raman spectral contrast. Additional clues were the optical and fluorescence contrast visible in bright-field and epifluorescence images of the measured area. By combining all available contrasts, it was possible to identify individual structures, delineate the areas (or 3D volumes) in the Raman maps where they were located in the cells and subsequently obtain averaged spectra from these areas for further analysis. In this way, averaged Raman signals from nuclei, nucleoli, lipid droplets, glycogen inclusions, VFs, VAs and cytoplasm were obtained.

The averaged Raman spectra also contained a Raman signal of water (a broad band between 3,000 and 3,750 cm^−1^) and thus it was possible to normalize them to the integral intensity of this band as an internal intensity standard [53]. In a similar way, it was possible to normalize measurements from different cells or different preparations when the Raman signal of water outside the cell served as an internal standard. Subsequently, the spectral contribution of water was subtracted from the intensity-normalized spectra.

The sets of resulting spectra belonging to different organelles or intracellular structures were compared with each other using the singular value decomposition (SVD) [53]. Spectra obtained from multiple cells or individual measurements were included in the joint SVD analysis of spectra extracted from individual structures to compare the spectral differences within and between groups using reduced representation. The SVD analysis decomposes a set of jointly analysed spectra into orthonormalized abstract spectral components referred to as subspectra S_j_, singular values W_j_ expressing their weights and loading coefficients V_ij_ expressing the representation of subspectrum S_j_ in a particular spectrum Y_i_ of the analysed set. If some spectra are similar to each other, their loading coefficients V_ij_ also take on similar values, i.e. they cluster in the reduced space of loading coefficients. Hence, cluster analysis can be used to group loading coefficients V_ij_ according to statistically significant similarities that may not be apparent at first glance. From the subspectra S_j_, it is then possible to read which spectral features distinguish the individual clusters from each other. However, this spectral information is relatively abstract because differences in specific Raman bands can be mapped to several subspectra S_j_. Instead, averaging the spectra within each cluster (i.e. their coefficients V_ij_) yielded statistically significant and meaningful typical spectra for each individual group. These spectra could be compared to each other in a straightforward way such as difference spectroscopy. The clustering approach also helped to identify outliers (i.e. objects not belonging to any spectral cluster) representing, e.g. incorrectly assigned structures.

## Results

### Temporal and spatial evolution of viral factories throughout infection

The first question was whether VFs undergo morphological and phase changes (e.g. as for RV) and/or relocate within the cellular context during infection (e.g. as for MRV). The progression of infection was followed by multiple imaging techniques in BHK-21 cell line stably expressing non-structural protein σNS tagged at the C-terminus with red fluorescent protein mCherry (σNS–mC). The tagged viral protein served as a convenient VF marker [35] after the cells were infected with ARV vaccine strain S1133.

Two-dimensional (2D) epifluorescence was used to identify virus replication sites and was complemented with concerted holotomographic microscopy during live-cell imaging, which provided cellular context, e.g. the proximity of organelles such as nuclei. Holotomographic microscopy reconstructs 3D images by measuring optical phase shifts caused by variations in cellular RI, using holographic transillumination from multiple angles. The resulting 3D RI maps are roughly proportional to the local mass density and provide label-free contrast for the visualization of intracellular structures such as nuclei, membranes and high-density phases like nucleoli or other biological condensates (see [35] for further methodological details). The RI maps can be subjected to 3D volume segmentation in a similar fashion to any other density maps, and the epifluorescence signal serves as a marker of biological activity, i.e. virus replication, helping to identify structures relevant to virus assembly.

The viral non-structural protein σNS is essential for virus replication and highly abundant in infected cells. It has been previously shown that the other highly abundant non-structural protein μNS is necessary (and sufficient, Fig. S2C) for the formation of the cytoplasmic globular inclusions [25] and for the recruitment of σNS into the VF [18]. In the absence of μNS, i.e. in the absence of viral infection, σNS is uniformly distributed in the cells that are stably expressing σNS–mC (Fig. S2A). Upon infection, virus-driven expression of unlabelled μNS leads to VF formation and recruitment of σNS–mC (expressed in the cell), resulting in the redistribution of the fluorescence signal into globular cytoplasmic inclusions and the appearance of multinucleated syncytia (Fig. S2B). Thus, formation of globular cytoplasmic inclusion containing σNS–mC is concomitant with infection and can be considered as marker for sites of viral replication and assembly.

Epifluorescence imaging of live cells revealed bright, mobile cytoplasmic spots of σNS–mC on a diffuse background as early as 9 hpi (Fig. 1a, b and Supplementary Video 1). At the early stage, cells exhibited a single nucleus from which σNS was excluded, being uniformly dispersed throughout the cytoplasm apart from bright, micron-sized mobile spots in certain cells (Fig. 1b). Given that σNS relies on expression of virally encoded μNS for VF formation and localization to VFs [18, 25] and that these bright and mobile fluorescent spots were absent from the uninfected cells (Fig. S2A), these areas were assigned to VFs and marked the onset of viral infection and morphogenesis.

**Fig. 1.**
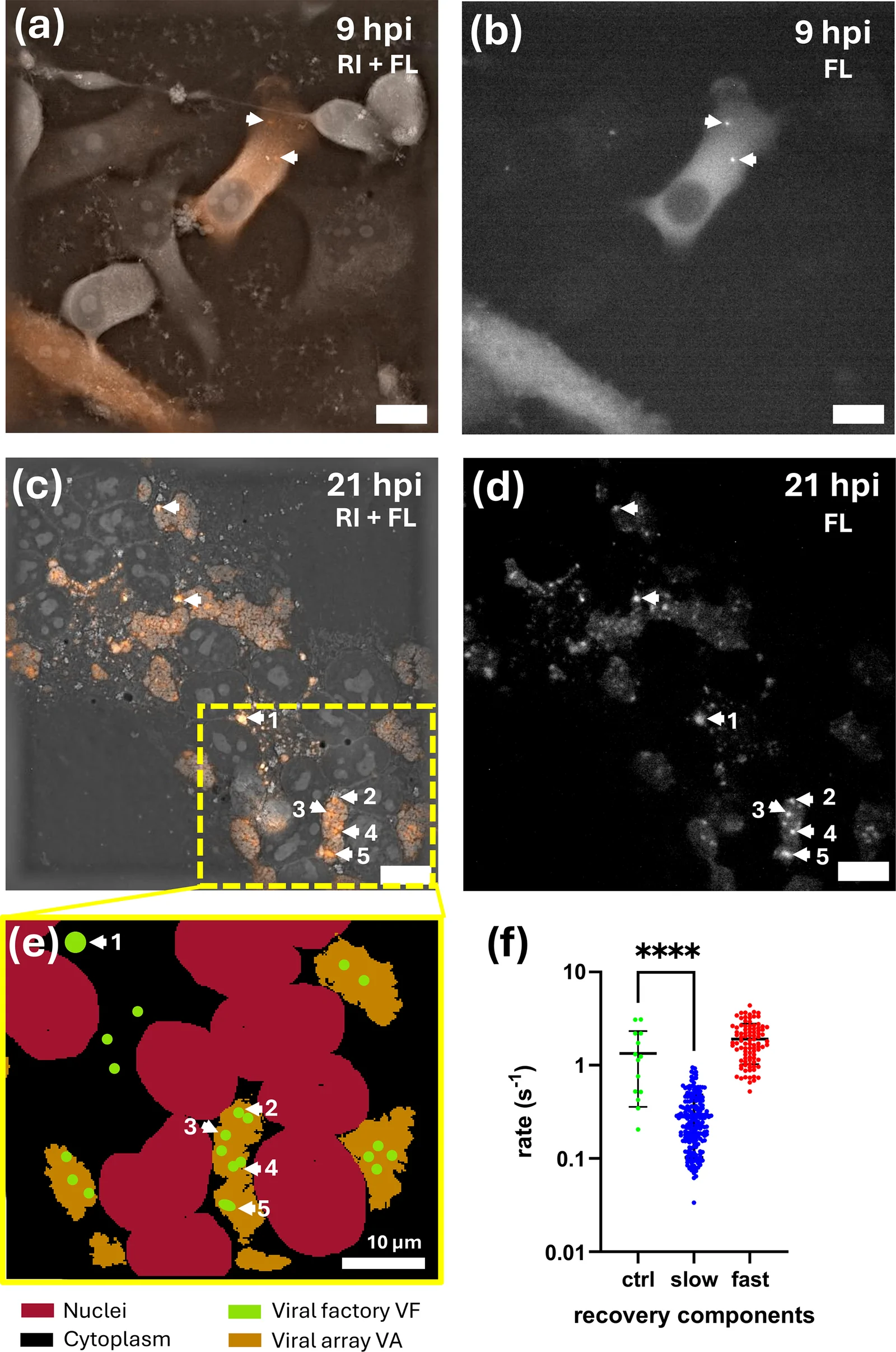
Fluorescence microscopy of BHK-21 cells stably expressing σNS–mC and infected with ARV. (**a**) Overlay of refractive index and mCherry fluorescence taken at an early infection stage (9 hpi). Mobile VFs are labelled with white arrows. (**b**) mCherry fluorescence showing globular VFs (white arrows) on the diffuse σNS–mC cytoplasmic background. Note that not all cells are stably transformed and exhibit fluorescence. (**c**) Overlay of RI and mCherry fluorescence taken at a late infection stage (21 hpi). Region of interest (ROI) for segmentation is indicated by a dashed box and selected bright fluorescent spots are indicated with white arrows and numbered for correspondence with other panels. (**d**) As in panel c but fluorescence channel only. Bright, mobile spots corresponding to VFs are highlighted by white arrows and numbered (within the ROI area defined in panel c). Scale bar 10 µm. (**e**) Segmentation of the RI map in panel c ROI with the selected bright fluorescent spots indicated with white arrows and numbers corresponding to those in panels c and d. The segmentation was based on the RI map, which reflects differences in mass density and spatial distribution of cellular components in the observed sample. The 3D RI map was built from 13 z-slices sampled at 400 nm using MIB [48]; red – nuclei; brown – VF/VA; green – sites of high fluorescence intensity assigned to σNS–mC. Scale bar 10 µm. (**f**) Statistical analysis of recovery rates; green – recovery of diffuse fluorescence in uninfected cells; blue – slow component; red – fast component.

When infection progressed into multinucleated syncytia, driven by production of fusogenic viral proteins, VFs remained mobile (15 hpi, Fig. S2D and Supplemetary Video 2). Tracking of the mobile globular VFs revealed random steps in all directions (Fig. S2E), ruling out directional transport along the cytoskeletal network. At this stage, some cells exhibiting diffuse fluorescence (i.e. non-infected or in early infection stages) coexisted with the syncytia.

At late stages of infection (detected here between 19 and 23 hpi), most cells were fused into multinucleated syncytia and the fluorescent areas became large and inhomogeneous with interspersed bright spots on a diffuse background (Fig. 1d, white arrows). The fluorescence was concentrated within areas of high RI (Fig. 1c), suggesting a composition different from that of the cytoplasm. RI-based volume segmentation of the holotomographic images revealed vast cytoplasmic regions with high RI (Fig. 1e, orange), often filling up most of the space between adjacent nuclei (Fig. 1e, red). The overlay of the segmented volume with the 2D fluorescence (Fig. 1e, green) revealed that σNS–mC concentration varied over these areas (‘hotspots’ indicated by white arrows). In comparison with earlier infection stages, the mobility of these fluorescent bright spots appeared restricted (compare Supplementary Videos 1, 2 versus 3), and the surrounding high-RI areas appeared solid-like (Fig. 1e, orange). This suggests that these areas are spatially inhomogeneous, with σNS undergoing partitioning into different regions exhibiting high and low σNS concentrations, respectively, and decreased mobility.

The inhomogeneous σNS distribution might reflect the coexistence of two phases with different fluidity, e.g. liquid and solid. However, σNS–mC remained freely exchangeable and mobile within all fluorescent areas throughout the infection, as shown by FRAP (Fig. S3A). The kinetics could be modelled either by one slow component or a combination of slow and fast components (Fig S3B). Rates of the fast component were comparable to those obtained for the diffuse σNS–mC signal from the cytoplasm (Fig. 1f), thus representing a residual cytoplasmic contribution due to bleaching of the cytoplasmic areas proximal to the smaller VFs. The slow component fluorescence recovery was significantly slower (~3-fold, Fig. 1f) and was assigned to σNS–mC diffusing within the liquid VFs. No significant changes in recovery kinetics were observed over the course of infection (Fig. S3C). Average fluorescence recovery after the bleaching was ~80% (Fig. S3A), indicating no substantial contribution from gel/solid phases with restricted diffusion. Hence, the liquid character of VFs does not change over the course of infection.

### Cellular context of viral replication

To gain further insight into the nature of VAs and to identify possible assembly intermediates within VFs, multiscale EM imaging was applied. First, we performed FIB-SEM to obtain a 3D view of the infected cells (chemically fixed sample, late stage of infection 23 hpi). Images revealed multiple denser inclusion sites in the cytoplasm of a multinucleated cell (Fig. 2, Supplementary Videos 4 and 5). The inclusions are of irregular ‘cloud’ shape (Fig. 2CE, brown) of the size ranging from a few to tens of micrometres, easily reaching the size of the nucleus but generally being excluded by the nuclear membrane and often filling spaces between adjacent nuclei and sometimes invading the membrane network of the ER (Fig. 2E, magenta). The images revealed granular arrays of round particles of roughly 50 nm diameter (Fig. 2B, highlighted brown in panel d) enclosing densely stained areas (Fig. 2BD, green). Within each array, multiple irregular oval-shaped denser spots at apparently random positions were identified and assigned tentatively to the fluid, σNS-enriched VFs as observed in the live-cell imaging (Fig. 1C). At this resolution, EM allowed to detect less stained pockets inside dense VFs, suggesting that despite their apparent fluidity, particles such as assembly intermediates may be present (Fig. 2D, light cyan areas and arrows in panel b).

**Fig. 2.**
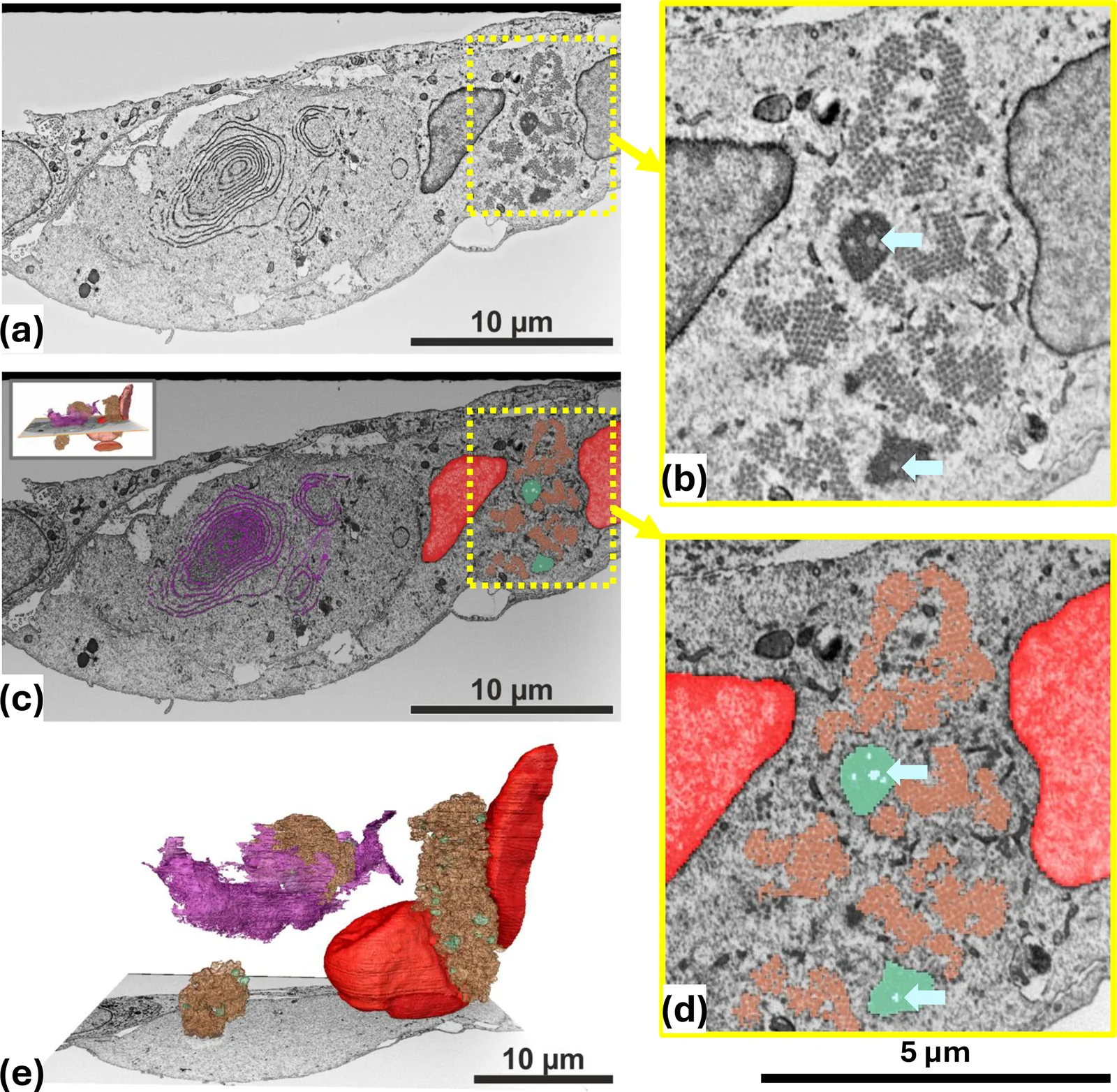
Reconstruction of VA/VF areas in the volume of an infected multinucleated cell based on FIB-SEM imaging at room temperature. (**a**) An example of one z-slice before segmentation. (**b**) zoomed area corresponding to the yellow dashed box in panel a. cyan arrows point to less dense spots (possibly virion-sized particles) inside the dense areas assigned to VFs. Panels (**c**) and (**d**) show segmentations of the areas in panels a and b, respectively: red – nucleus; violet – ER; brown – VA; green – VF; cyan spots and arrows pointing to less dense regions possibly containing virion-sized particles. The position of the z-slice within the reconstructed volume is indicated in the top left corner of panel c. (**e**) The 3D model of the observed volume. Segmentation was done in MIB [48], and the model was visualized in Amira (Thermo Fisher Scientific).

### Late viral factories contain replication intermediates and are surrounded by virion arrays

To reveal finer structural details, late VF areas within syncytia were subjected to serial section ET. A model reconstructed from ET data obtained at the location of apparent high density (Fig. 3a) on eight consecutive ultrathin sections revealed vast regular arrays of virus assembly intermediates and virions, designated here as VAs, assembled around the denser VFs (Fig. 3b, Supplementary Video 6). Few solitary virions can be seen in the VFs interior, and more are located closer to the VF periphery, suggesting that this is the site of virion assembly (Fig. 3b, c, red arrows). However, these larger particles do not accumulate within the dense VFs. This suggests that they are being rapidly excluded from the factory to the periphery, forming approximately hexagonally packed 3D arrays (Supplementary Video 6).

**Fig. 3.**
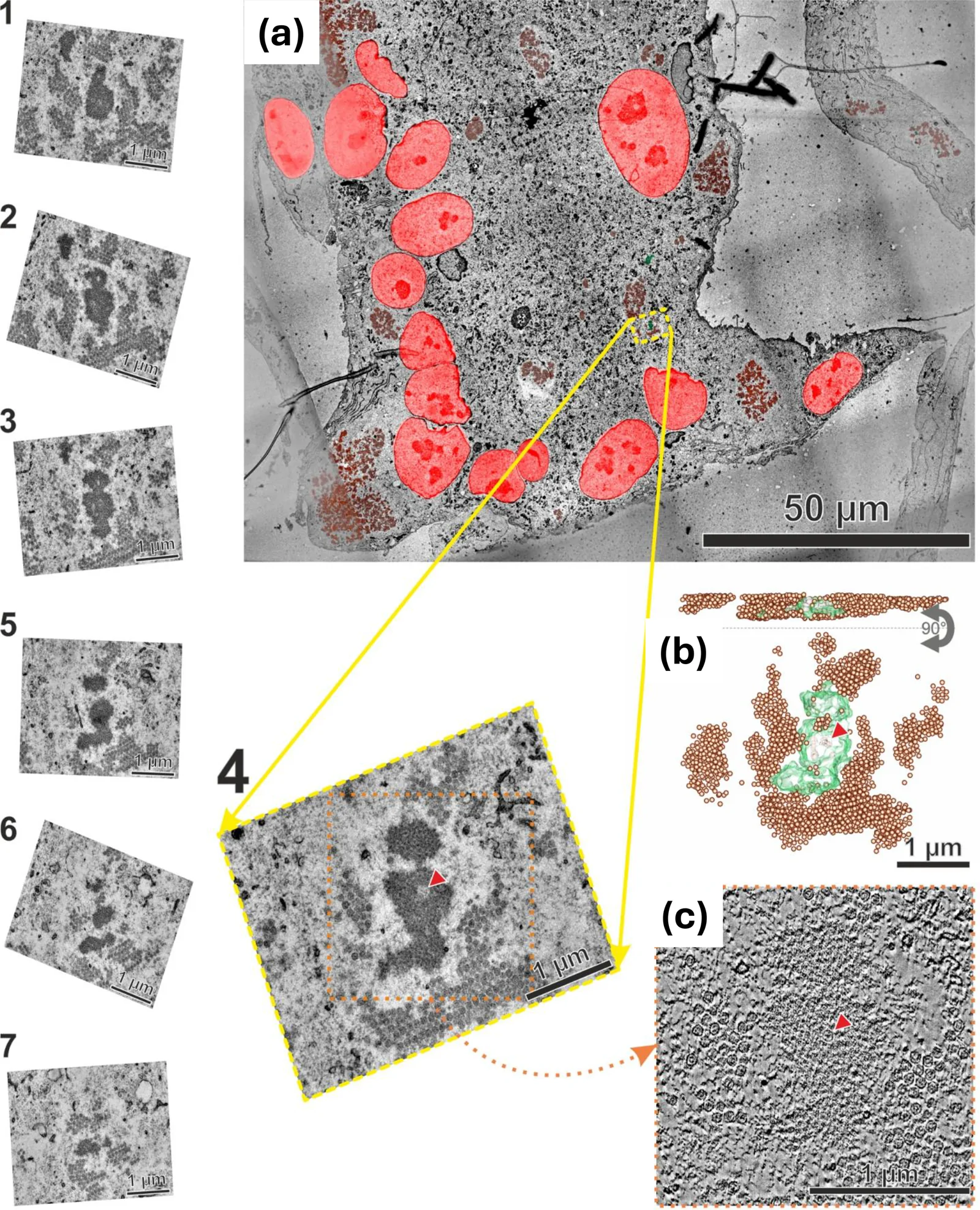
Serial section ET on ARV-infected BHK-21 syncytia after chemical fixation. (**a**) A portion of an infected (23 hpi) multinucleated cell visualized by TEM with cellular structures highlighted: red – nuclei; brown – VA areas; green – dense VF. Scale bar 50 µm. The yellow dashed rectangle shows the area selected for ET data collection and panels (1)–(7) the corresponding consecutive ultrathin sections. Scale bars 1 µm. Panel (4) shows the enlarged middle thin section, the red dashed box delimits the boundaries of the ET reconstructed volume. The red arrow points to the cavity within the densely stained volume attributed to VF. (**b**) 3D model of the ET volume delimited in panel (4). Brown – arrays of virion-sized particles (VAs); green – VF. The red arrow points to the cavity (white) within VF. (**c**) A middle slice of the final tomogram; the red arrow points to a virion-sized particle inside the cavity within VF. Scale bar 1 µm.

To reveal further structural details within VF, the infected cells were cryo-fixed, embedded in resin and sectioned. Thin sections were contrasted and examined by ET, which provided better resolution of fine VF structure and revealed smaller assembly intermediates with sizes corresponding to the size of ARV core ~30 nm, most likely representing SLPs (Fig. 4b). As seen above, very few fully assembled particles are visible inside VF in Fig. 4a). ET reconstruction of one ultrathin section demonstrated that the SLPs are arranged in a random fashion within the VF volume, as would be expected for a liquid phase (Fig. 4c). In contrast, most virion-sized particles are excluded from VF volume and accumulate around, favouring hexagonal packing.

**Fig. 4.**
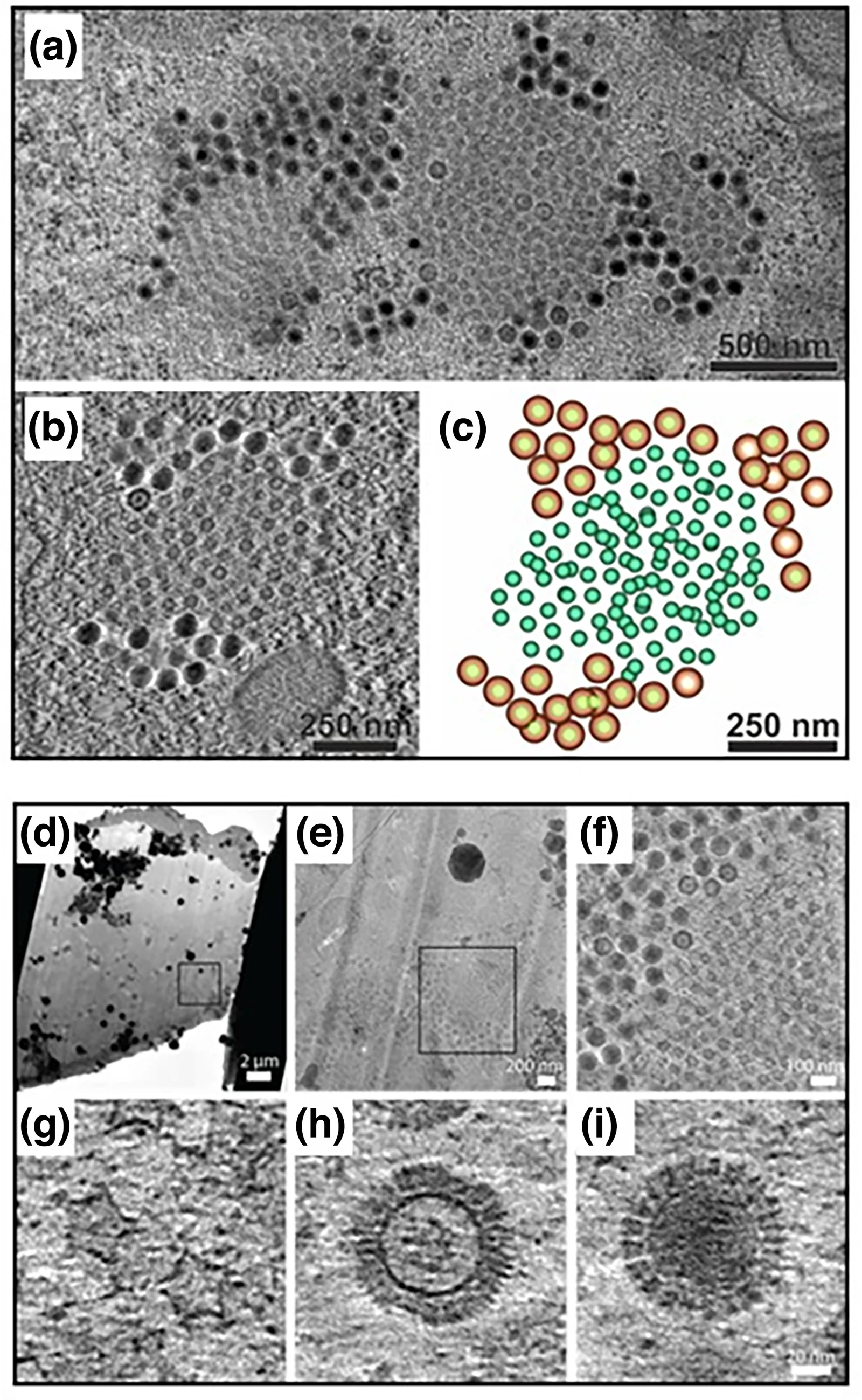
ET of one section of an ARV-infected BHK 21 cell fixed by cryo-fixation 23 hpi. (**a**) An example of a VA/VF area with VLPs (empty or partly empty) observable inside VF. Scale bar 500 nm. (**b**) Area of VA/VF selected for ET. Scale bar 250 nm. (**c**) A 3D model of the acquired tomogram showing small, core-like particles in green and mature-like VLPs in brown, and the internal green reflecting the apparent density, which may correspond to the RNA-filled core. Scale bar 250 nm. (**d-i**) Cryo-ET of an ARV-infected BHK 21 cell vitrified at 23 hpi directly on Au grids by plunge freezing, milled by cryo-FIB to prepare lamella. (**d**) Overview of the lamella in cryo-TEM. Scale bar 2 µm. (**e**) ROI corresponding to grey box in panel d that was subjected to cryo-ET. Scale bar 200 nm. (**f**) Section through the volume delimited in panel e showing the separation between VA and VF. Scale bar 100 nm. (**g**) Detail of SLPs inside VF. (**h**) Detail of half-filled, virion-sized double-layered particle (DLP) (**i**) Detail of a filled virion. Scale bar 20 nm for panels g–i is shown in panel i.

These conclusions were further supported by *in situ* cryo-ET performed on lamellae obtained by cryogenic FIB milling (Fig. 4d–i). This method preserved finer structural details of the intermediates and virus particles (Fig. 4g–i) and confirmed that the small particles are star-shaped, collapsed core assemblies (Fig. 4g) that are often seen in cells infected by reoviridae viruses [21, 38] and thus represent SLP assembly intermediates. While some of these stars exhibit internal densities that may represent RNA, no RNA-filled and expanded SLPs were found within the VF regions (Fig. 4f).

The native contrast afforded by cryo-ET allowed us to discern the presence of RNA inside virion-sized, double-layered particles (DLP) within VAs in the VF vicinity. These particles were classified as empty DLP, partially filled DLP (approximately half filled, Fig. 4h) and full virion (Fig. 4i). Analysis of all cryo-ET reconstructions identified a total of 448 DLPs (set as 100%), of which 312 appeared full (69.6%), 62 were classified as half-full (13.8%) and the remaining 74 (16.5%) were empty. No distinct types of particles were present at the very boundary between VF and VA regions. However, as shown by the average distance and the distributions of DLP distances from this boundary, the half-full DLPs localized significantly closer to the boundary than virions (Fig. S4). On the other hand, the localization of empty DLPs and virions was similar.

### Raman microscopy reveals distinct biomolecular composition of viral factories and arrays

The above results suggested that the mobile and fluid VFs in the early stages evolve into larger, inhomogeneous structures composed of fluid VFs embedded in less dense cytoplasmic VAs that exhibit diffuse σNS–mC staining. To gain more information about their respective biomolecular compositions, we employed CRM. CRM provides maps of vibrational spectra throughout the sample volume and hence allows label-free imaging, yielding contrast that is related to the biomolecular composition and concentration. Vibrational spectra contain so-called characteristic bands (or Raman signatures) that reflect the chemical structure of molecules and allow to discern the presence of certain biomolecules, such as proteins, lipids and nucleic acids, in complex mixtures [54] (Table S1). The relative intensities of the characteristic bands then provide information about the biomolecular composition.

To image VF by CRM, correlative fluorescence microscopy was used for navigation. Infected cells (9 hpi, early infection) were identified by the presence of round fluorescent VFs (Fig. 5a, b). The scanned area (Fig. 5b, yellow box) encompassed other cellular compartments (e.g. nuclei and cytoplasm) as well as portions of the extracellular medium. CRM spectral images were decomposed into three significant spectral components (Fig. S5). Based on the presence of characteristic bands (Table S1, Fig. S5), the three components corresponded to: (1) the medium, dominated by water (spatial distribution Fig. 5c); (2) nucleoprotein (Fig. 5d), assigned based on the presence of characteristic vibrations corresponding to proteins and nucleic acids; (3) lipids (Fig. 5e), assigned based on the presence of characteristic vibrations corresponding to fatty acids and phospholipid headgroups.

**Fig. 5.**
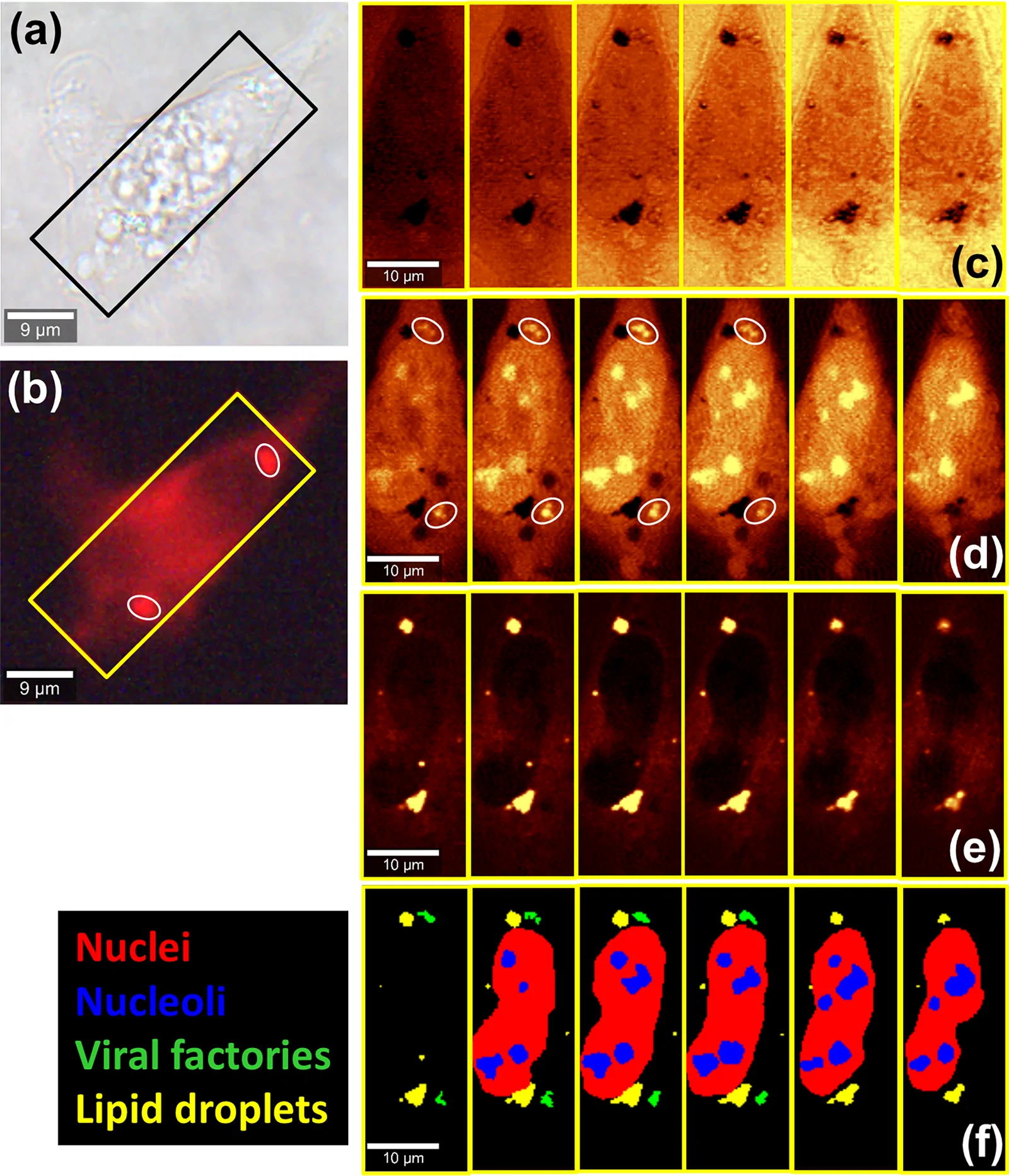
Raman imaging of infected cell syncytium (9 hpi) with two nuclei and image analysis based on spectral contrast. (**a**) Bright-field image. (**b**) mCherry fluorescence with ROI selected for Raman imaging delineated by a yellow box and localized σNS–mCherry. (**c–e**) Z-sections of true component intensities: (c) – medium; (d) – nucleoprotein; (e) – lipid; see Fig. S4 for the true component spectra. (**f**) Spatial masks based on the true component contrast and fluorescence, which delineate areas to extract and average Raman spectra of ROIs: lipid droplets – yellow; VFs – green; nuclei – red; nucleoli – blue. The extracted spectra are shown and compared in Fig. S5.

The lipid-dominated component is spatially localized into compact globules within the perinuclear space (Fig. 5e). These globules are void of water and nucleoprotein (appearing dark in Fig. 5c, d) and exhibit a Raman signature typical of lipid droplets [55]. The other two components contributed with different loadings to multiple cellular compartments, as illustrated by the nucleoprotein loadings contrasting nucleoli within nuclei and VFs within the cytoplasm, reflecting their higher biomolecular concentration. The regions assigned to VF (circled in Fig. 5d) coincide with σNS–mC fluorescence, demonstrating that the VF biomolecular composition differs from the surrounding cytoplasm, a clear hallmark of phase separation.

Using the contrasts derived from Raman spectra (Fig. 5c–e), the imaged volume was further segmented into 3D regions of interest (ROI3D, Fig. 5f) encompassing pixels with similar component loadings. Raman imaging and segmentation were also performed for late infection syncytia (Fig. 6), collecting data across wider regions with diffuse σNS–mC fluorescence between adjacent nuclei (Fig. 6b), which shall contain the high RI inclusions with VFs embedded within VAs (as seen in Fig. 1c-e). Spectral contrast within the internuclear areas was too weak to identify VAs using Raman signature alone. Hence, a combination of criteria was used to segment out internuclear regions that were likely to contain VAs (Fig. 6f, green): (1) high σNS–mC fluorescence, (2) nucleoprotein Raman signal contribution was higher than the cytoplasmic average (Fig. 6d) and (3) low lipid contribution (Fig. 6e). These putative VA regions mapped to the perinuclear areas (Fig. 6f, green) as expected based on RI (Fig. 1e) and EM (Fig. 2e) imaging, and thus the resulting average spectrum constitutes a good approximation to the true Raman signature of VAs, albeit with possible contamination of cytoplasmic signal. However, no finer structures corresponding to the embedded VFs could be resolved, presumably due to their small size and limited contrast within the late inclusions.

**Fig. 6.**
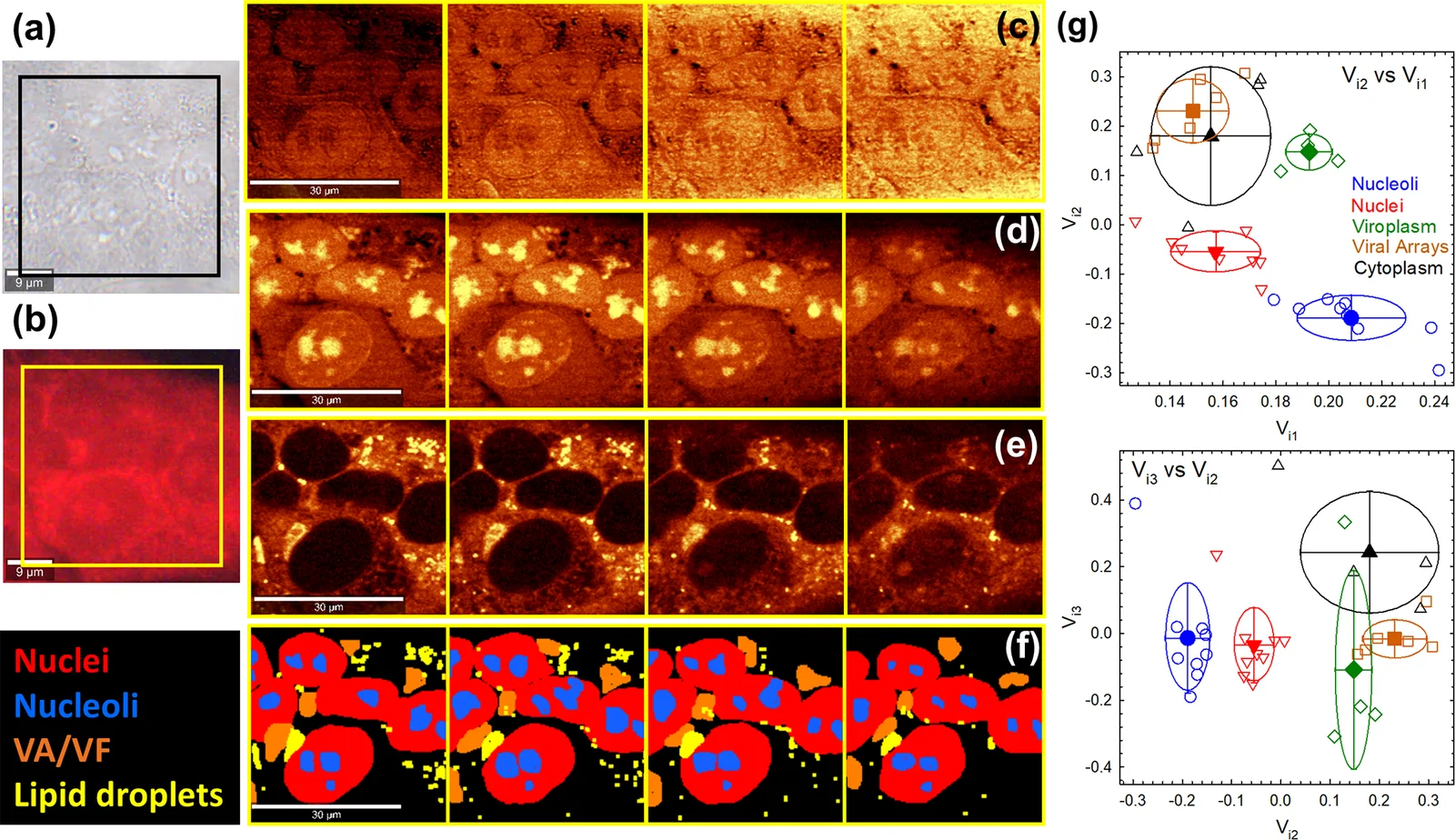
Raman imaging of a typical cell at the late stage of infection. (**a**) Bright-field image. (**b**) mCherry fluorescence with the ROI selected for Raman imaging delineated by a yellow box and localized σNS–mCherry. (**c–e**) Raman maps of true component intensities of lateral (xy) sections at four consecutive z-depths: C – medium; D – nucleoprotein-like; E – lipid, see Fig. S4 for the true component spectra. (**f**) Spatial segmentation based on the true component contrast and fluorescence: lipid droplets – yellow; dense cytoplasmic areas with σNS–mC fluorescence containing VAs and VFs – brown; nuclei – red; nucleoli – blue. (**g**) Clustering of loading factors for the extracted ROI spectra from different cells (see Fig. S6 for details of SVD analyses).

The average spectra of the segmented regions corresponding to nuclei (NUC), nucleoli (NCL), VFs, VAs and the cytoplasm (CYT, control spectra collected from the cytoplasm of uninfected cells) are shown in Fig. S6. At first glance, the VF spectrum resembles that of NCL, exhibiting also similar biomolecular concentration which is higher than that of the cytoplasm, as shown by the C-H stretching vibration intensity normalized with respect to the intensity O-H stretching of water (Fig. S6B). However, other differences are more subtle to decide whether, e.g. VF and VA regions have significantly different spectral signatures with respect to cell-to-cell variation.

One way to discern significant variation from noise is to reduce the spectra extracted from the segmented regions of different cells by SVD to components and cluster them using the corresponding loadings [56]. Based on the residual error and singular values, the variation across the data set was adequately described by three components [53] (Fig. S7, bottom row). While the loading coefficients obtained for the respective regions from different cells vary (Fig. S7, right column), they cluster into different regions of the 2D maps indicating significantly different biomolecular composition (Fig. 6g). The most significant spectral component S_1_ separates the dense VF and NCL from the rest of the spectrally segmented regions (CYT, NUC and VA). Component S_2_ further distinguishes NUC regions from VA and CYT, the latter two being differentiated only by the third, least significant component. Thus, the early VFs biopolymer concentration is higher, and composition differs from that of VA regions, as also shown by the normalized spectral difference (Fig. S6B). A significant difference at 784 cm^−1^ suggests the presence of a substantially larger amount of ssRNA in VFs than A-form dsRNA which contributes to a very small feature at 810 cm^−1^ [57]. As judged by higher intensity of many characteristic bands due to nucleic acid (Fig. S6B), the overall concentration of RNA is higher in VFs than in VAs.

In summary, early VFs, having biomolecular concentration higher than and composition different from the surrounding cytoplasm, are phase-separated cytoplasmic regions akin to the nucleolus being a phase-separated region of the nucleus (as also seen by CRM). On the other hand, the late-stage VA regions have biomolecular composition only slightly different from the cytoplasm but similar biomolecular density. Hence, VAs do not seem to have a clear signature of a separate homogeneous phase.

## Discussion

### Phase behaviour of viral factories and viral arrays

Unlike other members of *Reoviridae,* a single ARV protein μNS alone is sufficient to form VF-like inclusions when overexpressed in uninfected cells and recruits the RNA chaperone σNS [18]. The overexpressed μNS VF-like inclusions exhibit liquid droplet character and readily move around the cell, fuse and fission [35]. This liquid phase behaviour is also typical for the early stages of virus infection, as shown by their random mobility and FRAP, monitored via the recruited σNS–mCherry fluorescence (Fig. 1E and Supplementary Video 1). However, in the later stages, when virus infection spreads via cell–cell fusion and syncytia are formed, VF mobility is restricted by the surrounding VAs while the liquid-like character remains unaffected. This contrasts with RV infection, during which VFs undergo phase transition from the liquid to gel phase in later stages, a process which seems to be regulated by NSP5 hyper-phosphorylation [34, 58, 59]. Liquid phase behaviour was shown also for Blue tongue virus VFs [33] and the morphology of MRV VFs suggests similar behaviour [37, 60]; however, there is no direct proof of fluidity.

As shown by Raman spectral imaging, the liquid VF phase seems to have composition and biomolecular density similar to that of the nucleolus, reflecting its function as the site for RNA segment assortment that relies on σNS ribonucleoprotein assembly. The high biomolecular concentration may also facilitate assembly of the viral core. Interestingly, core dsRNA protein σA, which is recruited to VFs, was shown to localize also into the nucleolus using a nonclassical pathway [61]. Thus, VFs and nucleoli appear as similar condensates in which phase separation governs recruitment of components.

Later in infection, VAs engulf VFs and restrict their macroscopic mobility. VAs accumulate σNS to a lower degree than VFs (shown by fluorescence imaging (Fig. 1c, d)) and the protein remains microscopically mobile, as shown by unchanged FRAP kinetics (Fig. S3). While VAs exhibit biomolecular densities like nucleus and cytoplasm (Fig. S6A), their composition is slightly different from these compartments (Fig. 6g), namely having lower lipid content than the cytoplasm (Fig. S6). VAs are also significantly less dense than VFs and exhibit lower nucleic acid content (Fig. S6B). While virions appear hexagonally packed over short distances, they are interspersed by defects and vacancies, and most likely just fill the available cytoplasmic space and exclude larger structures such as membrane vesicles and compartments. Thus, formation of the VA phase is driven by high local concentration of virions that emanate from and mature at the VF interface. This contrasts with VF formation, which is driven by interactions between components (i.e. active recruitment) and results in a localized increase in biomolecular density.

Only a few solitary virions can be seen in the VFs interior, and likewise, no core particles are discernible within VAs. Thus, there is a clear boundary between VF and VA, and larger DLPs appear to be excluded from the fluid factories (Figs. 3 and 4) via the periphery to VA space. This may be due to the microscopic phase separation between the larger icosahedral virions and heterogeneous VF components, driven by their different sizes, as seen in systems composed of spheres of different diameters [62].

### Cellular context of virus assembly

The early VFs exhibit high but random mobility (Fig. S2C), which is not compatible with directional transport into a specific part of the cell. While later in infection VFs become less mobile, they appear randomly dispersed within the vast VAs due to being engulfed by the newly produced virions. In turn, VAs fill most of the cytoplasmic space between the adjacent nuclei (Fig. 1e and Fig. 2) within large syncytia. Hence, there is a clear difference between the morphogenesis of MRV, in which the early VFs serve as the site of core assembly, are located at the cell periphery, and then migrate towards the perinuclear space to mature before producing DLPs [37].

In early infection, ARV VFs are clearly separated in space from lipid droplets (Fig. 5), while in late infection, VFs are surrounded by the vast VAs, which penetrate the ER network and fill the perinuclear space (Figs 3 and 4). Thus, there is no clear direct involvement of lipid droplets and membranes in the VF formation, which is again different from the involvement of lipid droplets in the assembly of other members of *Reoviridae*. During MRV infection, lipid droplets act as carriers of the outer capsid proteins and associate with the VFs during their maturation [37]. Likewise, formation of reovirus replication factories was shown to depend on lipid droplets [23]. While lipids seem to be involved in different ways in the assembly of various viruses [58], their contribution to the morphogenesis of fusogenic orthoreoviruses seems limited.

### Assembly pathway

The early liquid VFs (Fig. 7a) exhibit only a limited number of virions around and contain assembly intermediates such as star-shaped SLPs (Fig. 4). These star-shaped structures appear empty or with minimal internal density (Fig. 4), incompatible with the presence of the complete set of genomic precursors. They resemble similar early assembly intermediates of the MRV [21] and the procapsid (SLP equivalent) of *Cystoviridae* bacteriophages [40]. Similar early intermediates were identified for other dsRNA viruses, and thus, VFs are most likely the sites of assembly initiation and core precursor assembly. However, unlike MRV VFs, which contain both SLPs and DLPs [21], ARV early VFs are almost completely void of virion-sized particles, suggesting that the assembly pathways of these two structurally related viruses are different [9]. ARV virion-sized particles seem to be excluded from VFs and accumulate as VA clouds in late infection (Fig. 4).

**Fig. 7.**
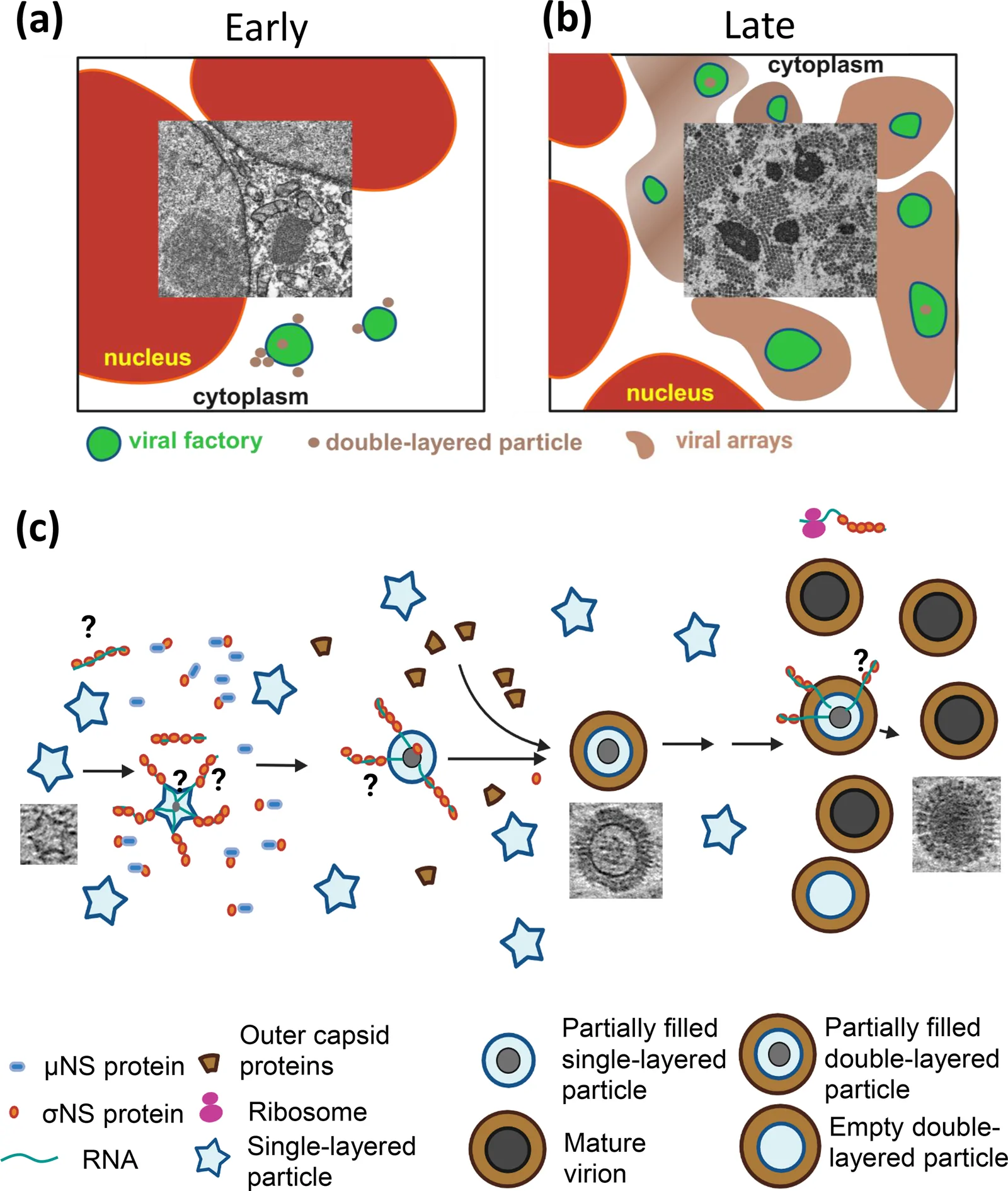
Cellular context and virus assembly pathway: (**a**) Schematics of mobile, early VFs (green) with virions (brown) egressing in the early syncytium. (**b**) Schematics of immobile late VFs (green) embedded in VAs (brown) with occasional virion-sized particles inside (brown spheres) within a multinucleated (nuclei – red) late syncytium. Insets show the electron micrograph on which the schematics were templated. (**c**) Proposed two-phase assembly pathway starting within the fluid, phase-separated VFs, where SLPs containing the hypothetical assortment complex are formed and then converted into partially filled DLPs either within the VFs or at the VF boundary and rapidly excluded into the VA area, where replication and packaging may continue to yield filled virions. Created in BioRender https://BioRender.com/djvmxfx.

Given that the observed SLPs contain little or no RNA and resemble the non-expanded, empty form of the bacteriophage procapsid [63] suggests that the RNA is packaged into these particles later. In the packaging mechanism of *Cystoviridae*, SLP selectively binds ssRNA segment precursors [64], which are then sequentially translocated into the particle by a packaging motor P4 ATPase [65] while the SLP inflates [40, 66]. The absence of partially inflated core intermediates and the packaging ATPase motor make the sequential mechanism of packaging unlikely. The absence of partially filled core intermediates was also noted for MRV and RV, suggesting that the packaging mechanisms of these viruses may be similar but differ from the bacteriophage sequential mechanism. Instead, their packaging and replication require RNA chaperones (ARV and MRV σNS, RV NSP2) that were shown to remodel RNA secondary structure and mediate specific RNA-RNA interactions between segment precursors [27, 32, 67–69]. This is considered a critical step in the recruitment of RNA precursors into the so-called ‘genome assortment complex’ that selects the exact number of segments to be incorporated into each core particle [31].

Raman spectral imaging suggests that VFs are rich in ssRNA, while fluorescence microscopy demonstrated that σNS is abundant in this phase (Fig. 1). Thus, most of the RNA within VFs is likely complexed with σNS, which has high affinity for ssRNA (and does not bind dsRNA) [27, 69] and may form helical filaments in the presence of long ssRNA [70]. However, filaments were not discernible in the cryo-EM images of VFs (Fig. 4), presumably due to limited contrast within the dense condensate. While no structurally distinct complex could be discerned within the interior of star-shaped SLPs, partial internal densities were detected in cryo-fixed samples in virion-sized DLPs (Fig. 4), suggesting that these particles may contain RNA representing the assortment complex or a replication intermediate. The partially filled DLPs, while occasionally found also in VFs, mostly accumulate within VAs but are localized closer to the boundary between VF and VA (Fig. S7), suggesting they represent replication and/or packaging intermediates. On the other hand, the empty and full DLPs are evenly distributed within VAs, suggesting that the former may be abortive products of core expansion and outer shell coating. Hence, while the outer capsid layer assembly may start within VF, replication seems to continue within virion-sized particles that are rapidly excluded into the surrounding VA zone. Given that σNS–mCherry fluorescence is also detected within VAs (Fig. 1c), it is plausible that these partially filled particles represent packaging intermediates with genomic ssRNA precursors sticking out through the vertices and complexed with σNS outside.

Based on the observations presented here, we propose a hypothetical assembly pathway (Fig. 7). The morphogenesis process relies on μNS formation of liquid VF into which σNS and viral structural proteins are recruited. The assembly starts within these VFs by formation of the hypothetical, compact RNA assortment complex via RNA–RNA interaction catalysed by σNS. This complex then nucleates assembly of the star-shaped core around most of the RNA, with the RNA sticking out of the capsid pores and being coated by σNS in filaments. This leads to the activation of the polymerases within the particle, which then replicate the genomic segments and drag the RNA into the core, acting as a molecular motor (RNA polymerase has been shown to be a powerful molecular motor, working against forces up to 14 pN [71]). During replication, the core expands, allowing for outer layer assembly, presumably still within VF. The resulting DLPs are rapidly excluded, most likely by size-driven phase separation, from the VF interior to the boundary layer between VF and VA, where replication and coupled packaging continue, as suggested by the presence of σNS within VAs. This is compatible with the absence of expanded and filled core particle from both VFs and VAs. This ‘maturation’ step within VAs is also supported by the observed gradient of partially filled DLPs within VAs (Fig. S4). As indicated in the schematics (Fig. 7, question marks), many of the steps and intermediates are still hypothetical, requiring more detailed structural as well as functional studies.

The proposed assembly mechanism clearly differs from the MRV assembly pathway, which relies on the transformation of factories from an early stage that contains cores and produces RNA to a late stage producing DLPs in association with lipid droplets [37]. Such functional and spatial segregation and lipid droplet involvement seem absent from ARV assembly, perhaps reflecting the fusogenic nature of this virus, which leads to gross morphological changes in the virus-induced syncytia, in which the remaining cytoplasm, as well as the ER network, are invaded by the vast VAs.

The factories described here are virion-producing factories, and the core particle precursors, clearly being void of dsRNA, do not produce any mRNA but are transformed into new infectious virions. This opens a question: where are the sites of viral ssRNA production? One possibility is that a small fraction of VFs that are embedded within vast VAs indeed produces cores filled with RNA and simply escaped detection during serial sectioning due to limited resolution. This situation would be similar to MRV, however, without the spatiotemporal segregation. An alternative mechanism would be that RNA is produced by the freshly replicated but VLP-like (double-layered) intermediates at the boundary between VF and VA, where σNS is also present to protect and concentrate the RNA into the adjacent VFs. It remains to be seen whether ARV DLPs can be transcriptionally active, a feature that has not been explored thus far.

## Supplementary material

10.1099/jgv.0.002350 Supplementary Material 1.

## Abbreviations

ARV: avian reovirus
BHK: baby hamster kidney
BTV: bluetongue virus
CRM: confocal Raman microscopy
DLP: double-layered particle
ER: endoplasmic reticulum
ET: electron tomography
FIB-SEM: focused ion-beam scanning electron microscopy
FRAP: fluorescence recovery after photobleaching
hpi: hour post-infection
LLPS: liquid–liquid phase separation
MRV: mammalian reovirus
NS: non-structural
RI: refractive index
ROI: region of interest
RT: room temperature
RV: rotavirus
SLP: single-layered particle
SVD: singular value decomposition
TEM: transmission electron microscopy
VA: viral array
VF: viral factory
VLP: virus-like particle
