# Supplementary material for: In situ multimodal imaging reveals compartmentalization of reovirus replication factories
Source: J Gen Virol. 2026 Oct 27;107(10):002350. doi: 10.1099/jgv.0.002350 (PMC13713037; doi:10.1099/jgv.0.002350)
Supplement: Supplementary file 1 [file jgv-107-02350-s001.pdf]

Supplementary Figures

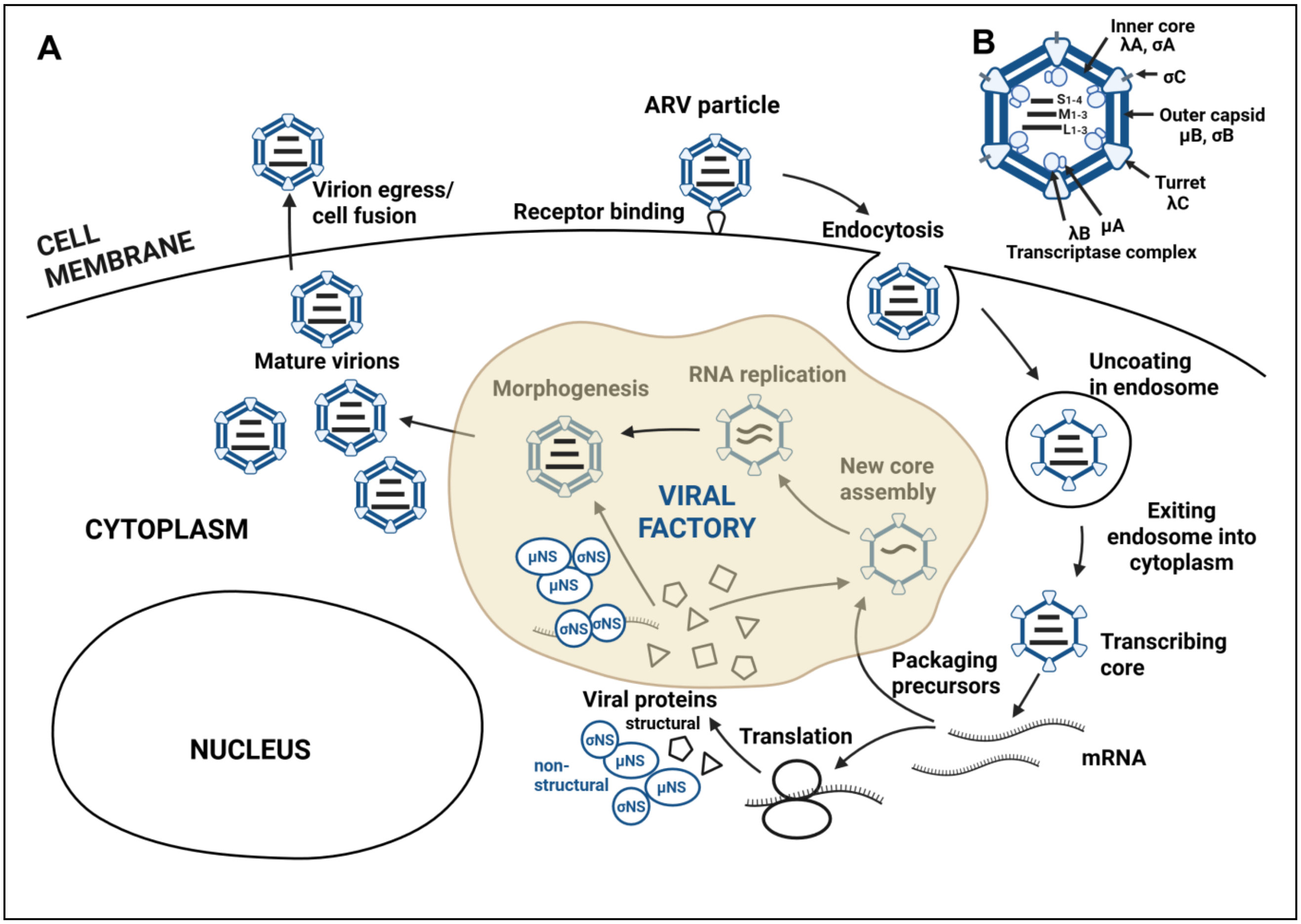

Figure S1. ARV replication cycle and structure. (A) Simplified schematics of ARV infection cycle (based on [1]). The processes thought to take place in the viral factories are presented on a beige background. (B) Schematics of ARV virion structure with the locations of the key structural proteins indicated by arrows. S, M and L denote small, medium, and large groups of dsRNA segments, respectively. Adapted from [2]. Created in BioRender. Durinova, E. (2026) https://BioRender.com/na70h38.

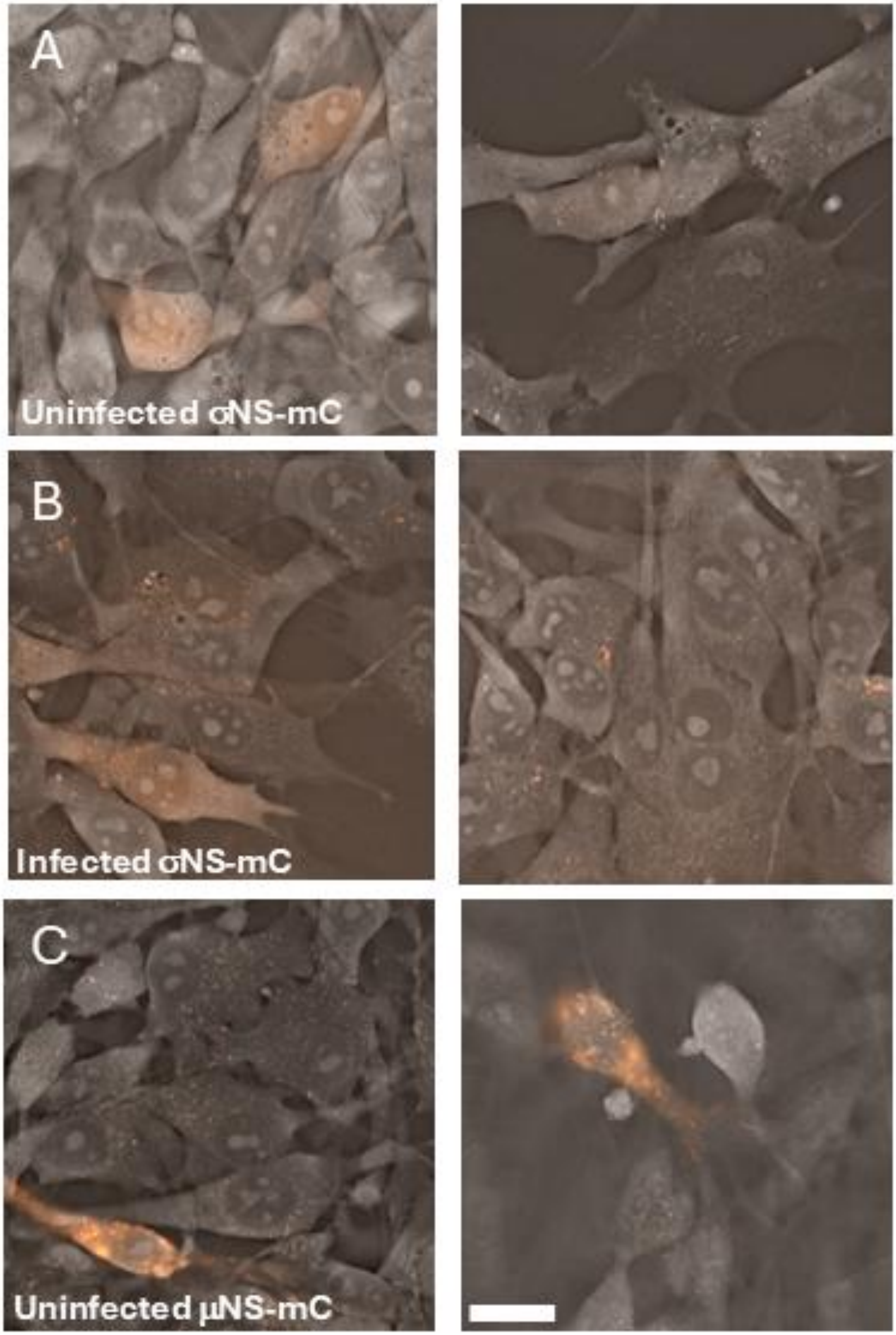
A
Uninfected σNS-mC
B
Infected σNS-mC
C
Uninfected µNS-mC

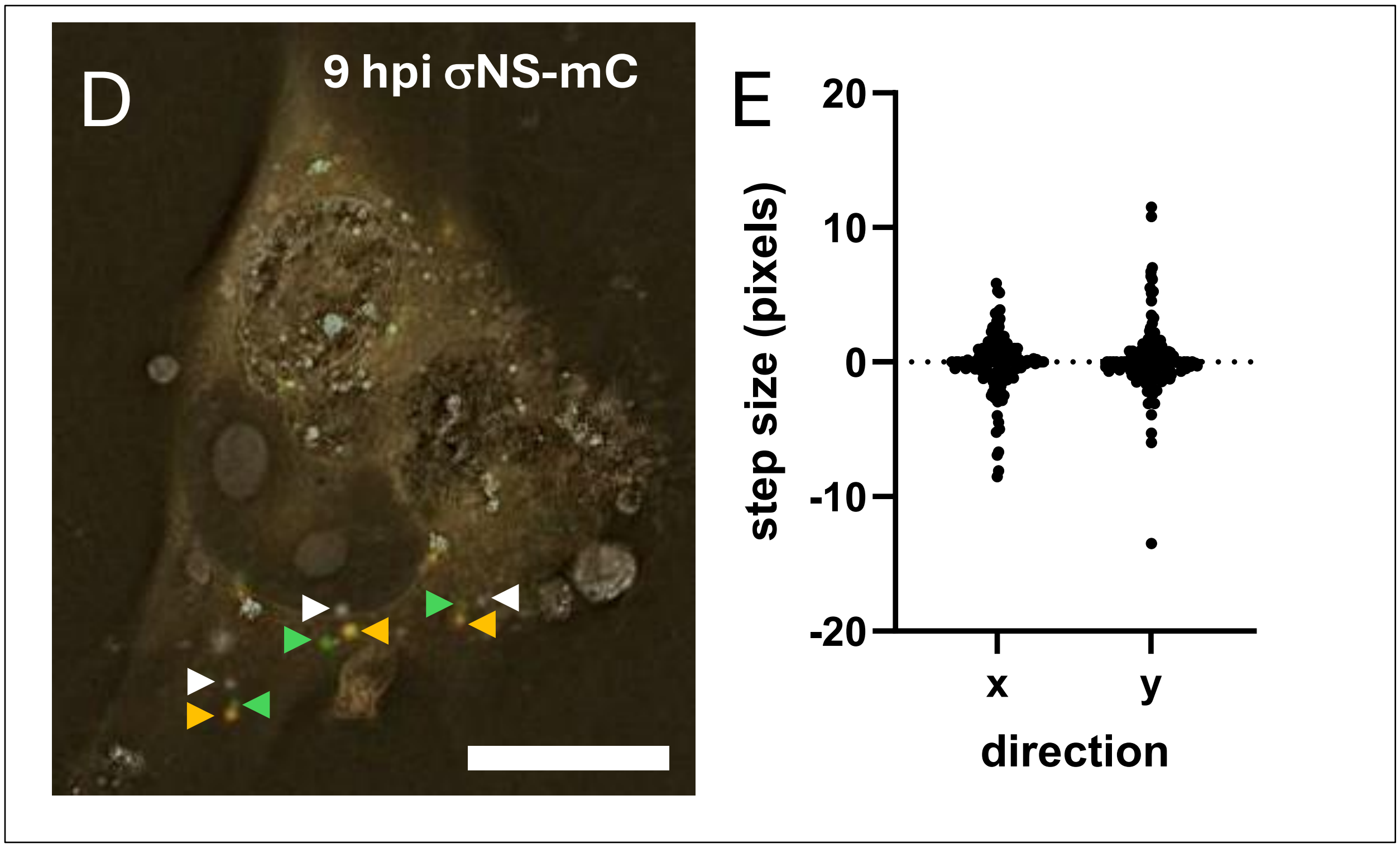

Figure S2. Imaging of BHK-21 cell cultures - overlay of mCherry tag (mC) epifluorescence (red) and refractive index central slice (grayscale). Scale bar 20 μm. (A) Diffuse distribution of σNS-mC epifluorescence in uninfected cells (two fields of view from separate experiments). Note that not all cells express σNS-mC and that the levels of fluorescence between cells vary. (B) Punctuate distribution of σNS-mC fluorescence in infected cells, i.e. in the presence of untagged μNS expressed from the virus genome (21h hpi, two fields of view from separate experiments). Note that not all fluorescent cells may be infected and thus may still exhibit diffuse distribution of σNS-mC fluorescence. However, formation of multinucleated syncytia is a marker of spreading infection and σNS-mC concentrates within cytoplasmic inclusions in those cells. (C) Punctuate distribution of μNS-mC fluorescence in uninfected cells stably transformed with μNS-mCherry fusion (two fields of view from different experiments). (D) Mobility of larger spots captured during time-lapse epifluorescence at 15 hpi overlayed in different colours (t = 0 min, orange, t= 3 min green, t = 8 min white) over the initial frame RI map (grayscale). Positions of fluorescence spots are indicated by arrows (t = 0 min, orange, t= 3 min green, t = 8 min white). Scale bar 20 μm. (E) Distribution of step increments along X and Y axes extracted from tracking VF objects in multiple time-lapse series.

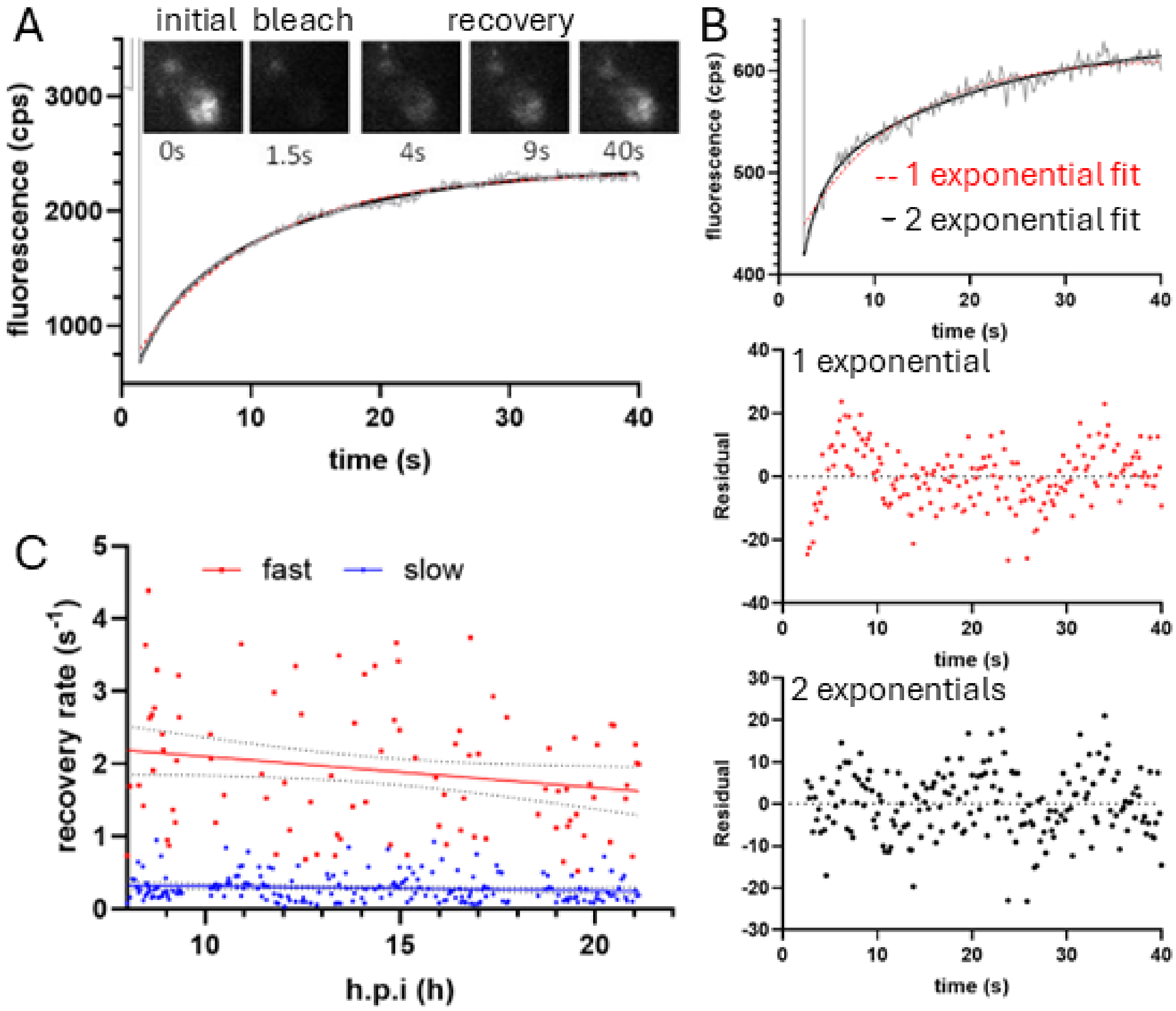

Figure S3. FRAP data analysis. (A) Fluorescence snapshots of the bleached spot (upper part) that illustrate the initial state, after the bleach pulse, and during the recovery phase. The lower part shows a typical recovery kinetics, which in this case can be approximated by one exponential component (red dashed line) while small contribution from a faster process is noticeable during the early recovery phase. (B) Top panel: An example of a more complex kinetics which necessitates the use of two components (fast and slow), as judged by significant departure of the one component fit from the experimental data and the non-random distributions of residuals (middle panel). The two-component fit (bottom panel) is sufficient to produce randomly distributed residuals. (C) Evolution of the recovery rates for the two components during the course of the infection. Recovery rates were extracted from FRAP data for individual spots at different times post-infection. Fast component – red; slow component – blue; dotted lines indicate 95% confidence intervals of the linear approximations (solid lines). Linear and non-linear fitting performed in Graph Pad Prism.

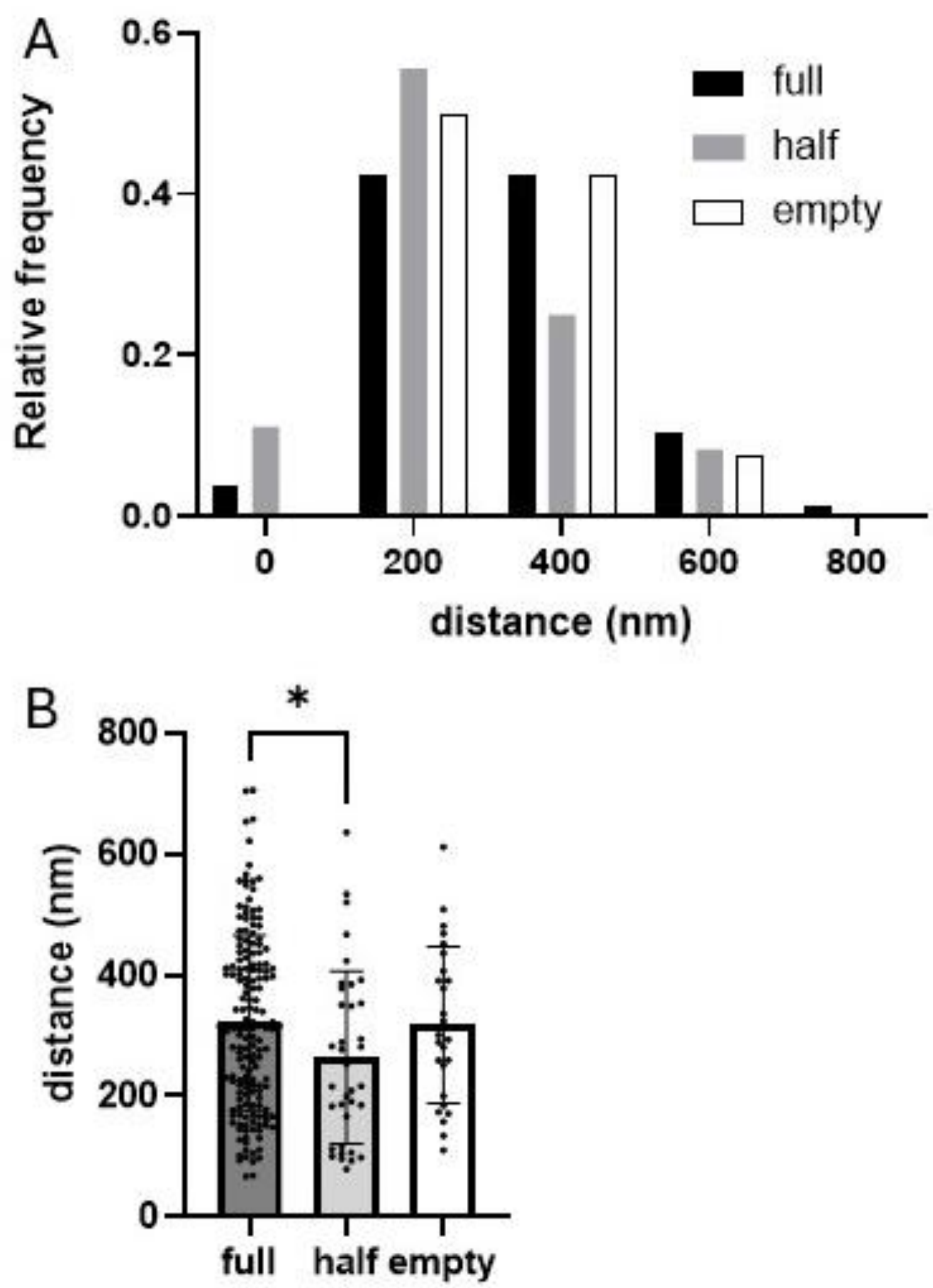

Figure S4. VLP distances from the VF boundary: (A) Normalized distance distributions for full, half-filled, and empty VLPs. (B) Average distances from the VF boundary with standard deviations and t-test significance. The distance is defined as the distance between particular VLP and its closest VLP located at the boundary.

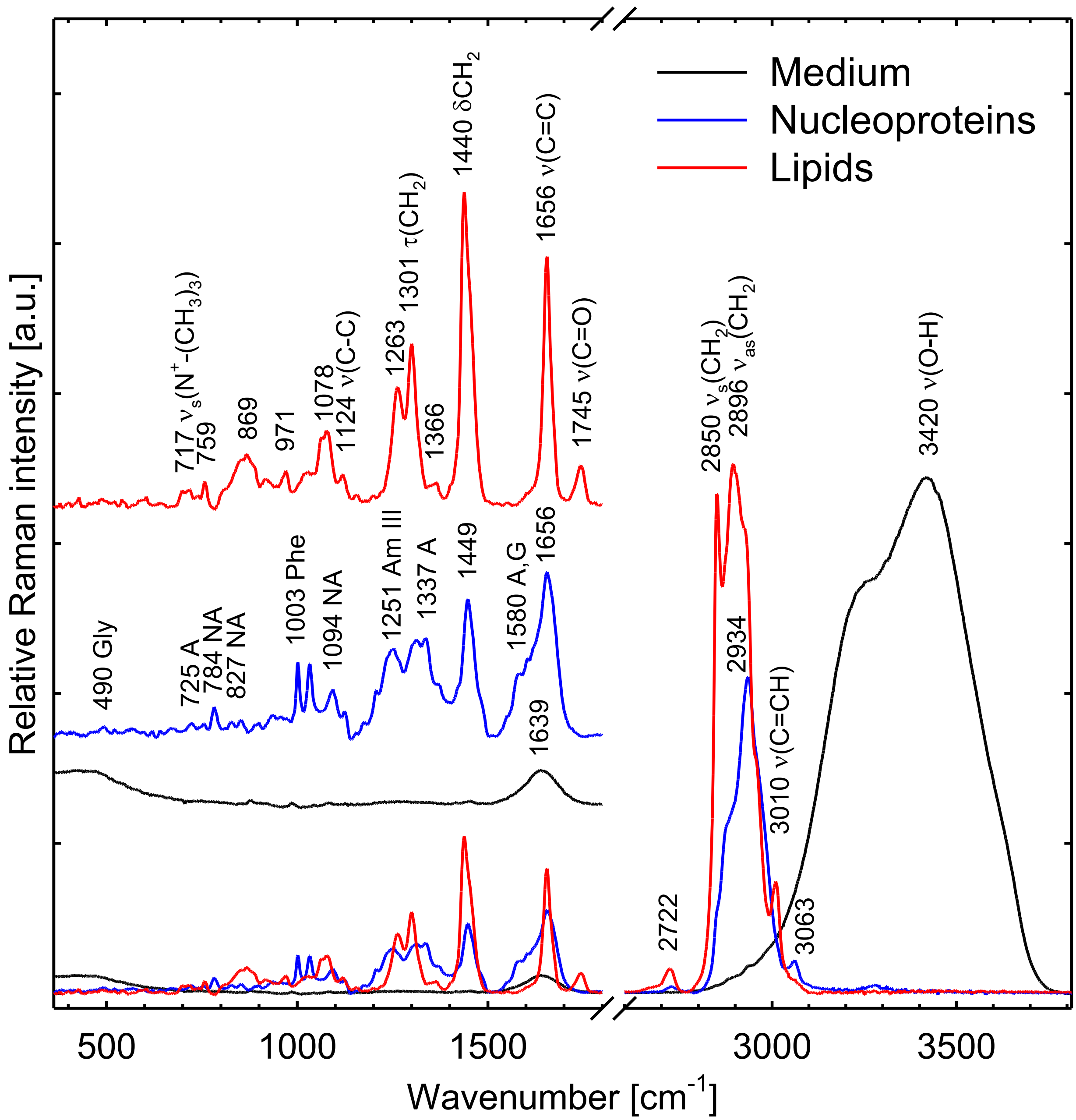

Figure S5. Raman spectra of three true components used for contrast generation. Characteristic regions of the spectra are inserted magnified twice and vertically shifted for better clarity. Black – medium dominated by water contribution at 1639 cm^{-1} (H-O-H bending mode) and the broad feature between 3000-3700 cm^{-1} corresponding to O-H stretching modes. Blue – nucleoprotein dominated by CH stretching mode 2934 cm^{-1} and lower frequency fingerprint modes of protein 1003 cm^{-1} (Phe ring) and nucleic acids 784 cm^{-1} (phosphodiester stretching), 1094 cm^{-1} (phosphoionic stretching) and 1337 cm^{-1} (nucleotide bases). Red - lipid dominated by CH stretching modes 2850 and 2896 cm^{-1} from fatty acids and lower frequency fingerprint modes of phospholipids at 1301 and 1440 cm^{-1}.

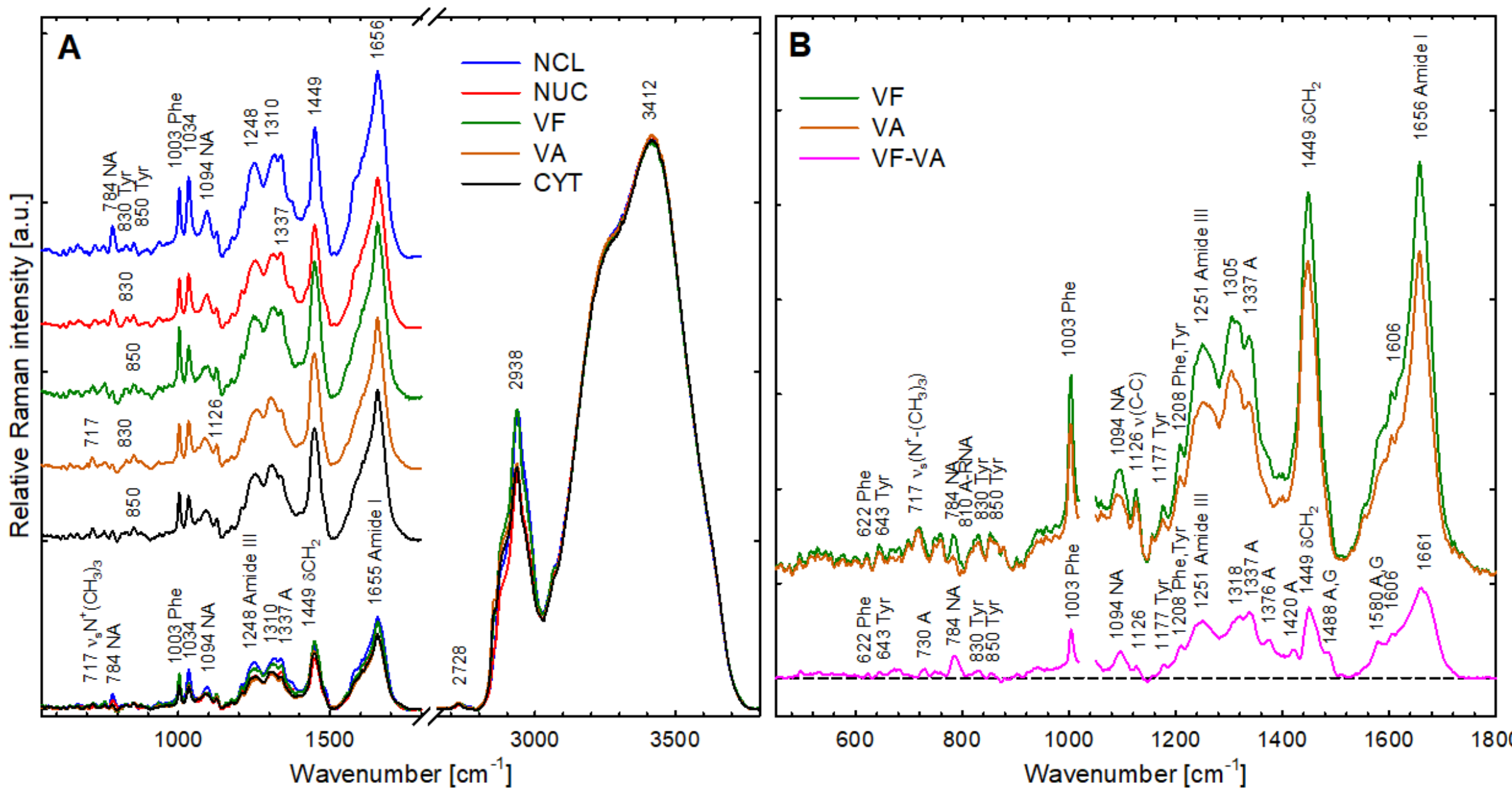

Figure S6: (A) Averaged Raman spectra extracted from ROI: Nucleoli (NCL, blue line), nuclei (NUC, red), viral factories (VF, green), viral arrays (VA, orange/brown), cytoplasm of uninfected cells (CYT, black). Spectra of the characteristic region (500-1800 cm^{-1}, left) and stretching C-H/N-H/O-H region are normalized on the integral intensity of the water O-H stretching band centred at 3412 cm^{-1} (right). The spectra in the characteristic region are magnified twice and vertically shifted for clarity. NA – nucleic acids, A – adenine, Phe – phenylalanine. (B) Direct comparison between the normalized spectra from VF and VA volumes was performed using digital difference spectrum of the fingerprint region (400-1800 cm^{-1}). Raman difference spectrum (bottom, magenta) between viral factories (VF, green) and viral arrays (VA, orange/brown), with prominent vibrations labelled. The overall positive values in the difference spectrum are due to intensity normalization and reflect the higher overall concentration of biomolecules within VFs compared to VAs.

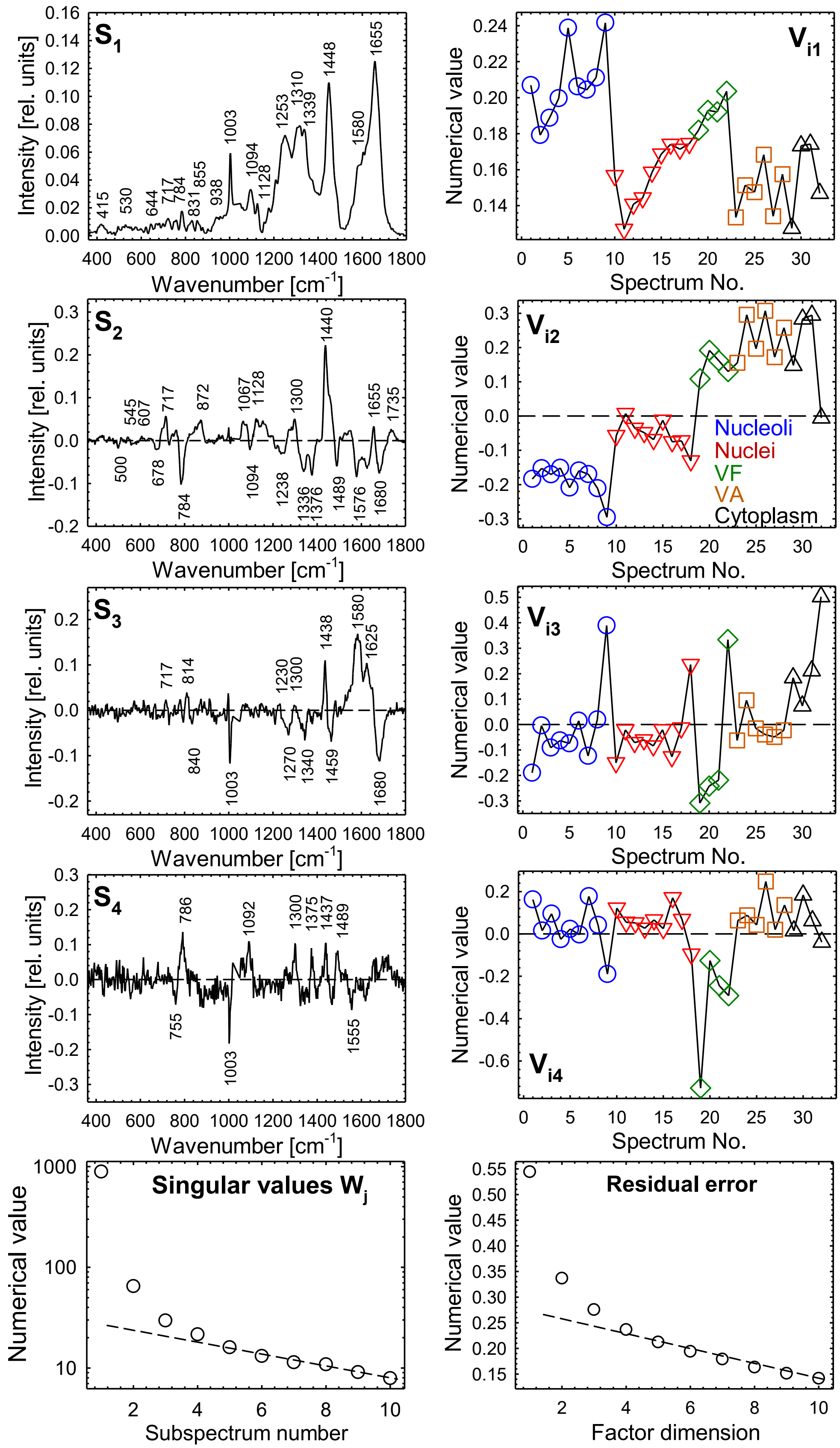
S1
Intensity [rel. units]
Wavenumber [cm-1]
415
530
644
717
784
831
855
938
1003
1094
1128
1253
1310
1339
1448
1580
1655
Vi1
Numerical value
Spectrum No.
S2
545
607
717
872
1067
1128
1300
1440
1655
1735
500
678
784
1094
1238
1336
1376
1489
1576
1680
Vi2
Nucleoli
Nuclei
VF
VA
Cytoplasm
S3
717
814
1230
1300
1438
1580
1625
840
1003
1270
1340
1459
1680
Vi3
S4
786
1092
1300
1375
1437
1489
755
1003
1555
Vi4
Singular values Wj
Numerical value
Subspectrum number
Residual error
Factor dimension

Figure S7. Joint SVD analysis of Raman spectra extracted from regions identified as nuclei (red triangles), nucleoli (blue circles), viroplasm (green diamonds), viral arrays (orange/brown squares), and cytoplasm (black triangles) in 3D Raman maps of nine different cells in the late stage of infection. The analysis was performed only in the spectral region of characteristic vibrations on spectra normalized to the integral intensity of the stretching vibrations of water. The magnitudes of the singular values and the residual errors indicate that the spectral variability of all analyzed spectra can be described with sufficient accuracy by three linearly independent components, while the fourth component (S_4) contributes only marginally. According to coefficient V_{i1}, the SVD analysis reveals that the density of organic matter in viral arrays is comparable to the density in nuclei (excluding nucleoli), which is, however, lower than the density of nucleoli alone. On the other hand, according to the coefficient V_{i2} of this analysis, viral arrays have a relatively higher proportion of lipids than nuclei or nucleoli. In the differential spectral component S_2, which emphasizes this difference, the typical Raman bands of lipids (717, 1300, 1440, 1655, 1735 cm^{-1}) contribute more to the spectra of viral arrays and, on the other hand, the Raman bands characteristic of proteins and nucleic acids (678, 784, 1003, 1238, 1336, 1376, 1576 cm^{-1}) contribute more to the spectra of nucleoli and nuclei. (see Table S1 below for assignments and references). VA and CYT regions segregate based on the least significant third component (Fig. 3G, right panel), which suggests that CYT regions might have a slightly higher contribution of lipids (positive V_{i3} loadings and positive peaks at 717 and 1438 cm^{-1}) while VAs are richer in protein (negative V_{i3} and negative peaks at 1003, 1270 and 1459 cm^{-1}).

Table S1. Wavenumbers of the major bands identified in the Raman spectra of viral factories and their assignment to the basic biomolecules.

Wavenumber [cm^{-1}]	Biomolecule	Assignment	References
478–480	Polysaccharides	Glycogen	[3]
620–622	Proteins	Phe	[4, 5]
642	Proteins	Tyr	[4, 6]
715–717	Phospholipids	$\nu_s(N^+–(CH_3)_3)$	[7, 8]
721–730	Nucleic acids	Ring breathing of A	[8]
750–758	Nucleic acids; Proteins	Ring breathing of T; Ring breathing of Trp	[4, 9]
782–786	Nucleic acids	Phosphodiester O–P–O	[8]
810	RNA A-form	Phosphodiester O–P–O	[8]
820-835	DNA B-form	Phosphodiester O–P–O	[8]
830	Proteins	Tyr Fermi doublet	[4, 6]
850–853	Proteins; Polysaccharides	Tyr; Glycogen	[3, 4]
936–939	Proteins; Polysaccharides	Glycogen	[3]
1002–1005	Proteins	Phe	[4, 5]
1034	Proteins	Phe; metal complexes of pyrophosphates	[4, 5]
1084–1086	Polysaccharides	Glycogen	[3]
1095–1100	Nucleic acids	Phosphodioxy $\nu_s(PO_2^-)$	[8]
1127–1130	Lipids; Polysaccharides	ν(C–C); Glycogen	[3, 7]
1156	Proteins	ν(C–C); ν(C–N); $\delta(CH_3)$	[10]

1175–1177	Proteins	Tyr	[4, 6]
1208	Proteins	Phe; Tyr	[4-6]
1254–1260	Proteins; Lipids	Amide III	[11]
1300–1302	Lipids	$\tau(CH_2)$	[7]
1330–1340	Nucleic acids; Polysaccharides; Proteins	Trp; A	[4, 8]
1442–1448	Lipids; Proteins	$\delta(CH_2)$	[4, 12]
1580–1585	Lipids; Proteins	Phe; Trp	[4, 5]
1623	Proteins	Trp	[8]
1653–1656	Lipids; Proteins	$\nu(C{=}C)$; Amide I	[4, 7]
1739–1742	Lipids	$\nu(C{=}O)$ in triacylglycerols	[7]
2558	Proteins	$\nu(S–H)$	[4, 13]
2725	Lipids		[7]
2850	Lipids	$\nu_s(CH_2)$ in triacylglycerols	[7]
2880–2885	Lipids	$\nu_{as}(CH_2)$ in triacylglycerols	[7]
2930–2936	Lipids	$\nu_s(CH_3)$ in triacylglycerols and membrane lipids	[7]
3060–3065	Lipids	$\nu(C{=}CH)$	[7]

ν – stretching mode; δ – deformation mode; τ – twisting mode; Tyr – tyrosine, Trp – tryptophan; Phe – phenylalanine; A – adenine; G – guanine; C – cytosine; T – thymine; U – uracil

Supplementary note on Raman spectral image contrast interpretation

In addition to biomolecules, water contributes significantly to Raman spectra in a high frequency region (above 3100 cm^{-1}, where stretching of O-H bonds dominates) while organic molecules (incl. biopolymers) have characteristic vibrations corresponding to aliphatic C-H stretching between 2800-3100 cm^{-1}. The intensity ratio of these two spectral features is a good measure of spatially

resolved biomolecular concentration within cells [14]. Thus, Raman imaging can detect formation of phases with different biomolecular composition and densities, such as viral factories, which exhibit higher biomolecular density [2, 15]. Raman spectral signatures of cell components are complex and, unlike those of purified biomolecules or viruses [16], are hard to interpret [2]. Additionally, cell-to-cell variability makes direct comparison of individual spectra difficult. As with other large data sets, the spectral complexity and variability can be reduced with the help of true component decomposition, which is a variant of singular value decomposition or principal component analysis, of Raman spectra collected over large 3D volumes from multiple cells (or syncytia in late infection stages). The decomposition yields several component spectra and their contribution to each sampled point in space (loading coefficients). The loadings are used as contrasting variables for segmentation of the imaged volumes, i.e., they can delineate areas with significantly different biomolecular compositions. The presence and intensities of the specific characteristic bands then provide additional information about the biomolecular composition of these phases, e.g., whether lipids or nucleic acids are present.

Supplementary Videos

Video 1. BKH-21 cells 9 hpi showing mobility of fluorescently labelled VFs (20 s time lapse). Overlay of mCherry fluorescence (red) and RI (grayscale) focal plane slice.

Video 2. BKH-21 cells 15 hpi showing mobility of fluorescently labelled VFs (30 s time lapse). Overlay of mCherry fluorescence (red) and RI (grayscale) focal plane slice.

Video 3. BKH-21 cells 21 hpi showing mobility of fluorescently labelled VFs/VAs (10 s time lapse). Overlay of RI focal plane slice (grayscale) and mCherry (red) fluorescence.

Video 4. Animation of FIB-SEM 3D volume reconstruction, sectioning, and segmentation. The left panel shows surface representation and segmentation of late viral factories (green) within viral arrays (brown) and the nucleus (red). The bottom section shows segmented virions (brown dots). The right panel shows segmented serial sections through the volume.

Video 5. Animation of serial tomography 3D volume reconstruction and segmentation in the context of multinucleated syncytium (nuclei - red, ER network - magenta. The left panel shows surface representation and segmentation: VF – green, VA - brown. The right panel shows segmented serial sections through the volume.

Video 6. Tomographic reconstruction of a section containing viral arrays. 3D yields a packing model for VLPs. Green – segmented virions within arrays. Virions in the detailed subsection are colour-coded according to the plane to highlight the close, approximately hexagonal packing.

Supplementary references

1. **Benavente J, Martinez-Costas J**. Avian reovirus: structure and biology. *Virus Res* 2007;123(2):105-119.
2. **Durinova E, Mojzes P, Bily T, Franta Z, Fessl T et al.** Shedding light on reovirus assembly-Multimodal imaging of viral factories. *Adv Virus Res* 2023;116:173-213.
3. **Wiercigroch E, Szafraniec E, Czamara K, Pacia MZ, Majzner K et al.** Raman and infrared spectroscopy of carbohydrates: A review. *Spectroc Acta Pt A-Molec Biomolec Spectr*, Review 2017;185:317-335.
4. **Tuma R**. Raman spectroscopy of proteins: from peptides to large assemblies. *Journal of Raman Spectroscopy*, Review 2005;36(4):307-319.
5. **Hernández B, Pflüger F, Kruglik SG, Ghomi M**. Characteristic Raman lines of phenylalanine analyzed by a multiconformational approach. *Journal of Raman Spectroscopy* 2013;44(6):827-833.
6. **Hernandez B, Coic YM, Pfluger F, Kruglik SG, Ghomi M**. All characteristic Raman markers of tyrosine and tyrosinate originate from phenol ring fundamental vibrations. *Journal of Raman Spectroscopy*, Article 2016;47(2):210-220.
7. **Czamara K, Majzner K, Pacia MZ, Kochan K, Kaczor A et al.** Raman spectroscopy of lipids: a review. *Journal of Raman Spectroscopy*, Review 2015;46(1):4-20.
8. **Kruglik SG, Royo F, Guigner JM, Palomo L, Seksek O et al.** Raman tweezers microspectroscopy of circa 100 nm extracellular vesicles. *Nanoscale*, Article 2019;11(4):1661-1679.
9. **Benevides JM, Juuti JT, Tuma R, Bamford DH, Thomas GJ**. Characterization of subunit-specific interactions in a double-stranded RNA virus: Raman difference spectroscopy of the φ6 procapsid. *Biochemistry* 2002;41(40):11946-11953.
10. **Tuma R, Bamford JHK, Bamford DH, Thomas GJ**. Structure, interactions and dynamics of PRD1 virus. 2. Organization of the viral membrane and DNA. *Journal of Molecular Biology*, Article 1996;257(1):102-115.
11. **Maiti NC, Apetri MM, Zagorski MG, Carey PR, Anderson VE**. Raman spectroscopic characterization of secondary structure in natively unfolded proteins: alpha-synuclein. *Journal of the American Chemical Society*, Article 2004;126(8):2399-2408.
12. **Wu H, Volponi JV, Oliver AE, Parikh AN, Simmons BA et al.** In vivo lipidomics using single-cell Raman spectroscopy. *Proceedings of the National Academy of Sciences of the United States of America* 2011;108(9):3809-3814.
13. **Deniz E, Schmidt-Engler JM, Ulrich K, Oberle M, Wille G et al.** SH-It happens: S-H bonds as intrinsic 2D-IR labels in proteins. *J Chem Phys*, Article 2022;157(13):9.
14. **Palacky J, Mojzes P, Bok J**. SVD-based method for intensity normalization, background correction and solvent subtraction in Raman spectroscopy exploiting the properties of water stretching vibrations. *Journal of Raman Spectroscopy* 2011;42(7):1528-1539.
15. **Haller CJ, Acker J, Arguello AE, Borodavka A**. Phase separation and viral factories: unveiling the physical processes supporting RNA packaging in dsRNA viruses. *Biochem Soc Trans* 2024;52(5):2101-2112.

16. **Nemecek D, Stepanek J, Thomas GJ, Jr.** Raman spectroscopy of proteins and nucleoproteins. *Curr Protoc Protein Sci* 2013;Chapter 17:Unit17 18.
